# Host Receptors of Influenza Viruses and Coronaviruses—Molecular Mechanisms of Recognition

**DOI:** 10.3390/vaccines8040587

**Published:** 2020-10-06

**Authors:** Nongluk Sriwilaijaroen, Yasuo Suzuki

**Affiliations:** 1 Department of Preclinical Sciences, Faculty of Medicine, Thammasat University, Pathumthani 12120, Thailand; 2 School of Pharmaceutical Sciences, University of Shizuoka, Shizuoka, Shizuoka 422-8526, Japan; 3 College of Life and Health Sciences, Chubu University, Kasugai, Aichi 487-8501, Japan

**Keywords:** influenza viruses, coronaviruses, host range, spikes, receptors, sialyl glycans, protein receptors

## Abstract

Among the four genera of influenza viruses (IVs) and the four genera of coronaviruses (CoVs), zoonotic αIV and βCoV have occasionally caused airborne epidemic outbreaks in humans, who are immunologically naïve, and the outbreaks have resulted in high fatality rates as well as social and economic disruption and losses. The most devasting influenza A virus (IAV) in αIV, pandemic H1N1 in 1918, which caused at least 40 million deaths from about 500 million cases of infection, was the first recorded emergence of IAVs in humans. Usually, a novel human-adapted virus replaces the preexisting human-adapted virus. Interestingly, two IAV subtypes, A/H3N2/1968 and A/H1N1/2009 variants, and two lineages of influenza B viruses (IBV) in βIV, B/Yamagata and B/Victoria lineage-like viruses, remain seasonally detectable in humans. Both influenza C viruses (ICVs) in γIV and four human CoVs, HCoV-229E and HCoV-NL63 in αCoV and HCoV-OC43 and HCoV-HKU1 in βCoV, usually cause mild respiratory infections. Much attention has been given to CoVs since the global epidemic outbreaks of βSARS-CoV in 2002–2004 and βMERS-CoV from 2012 to present. βSARS-CoV-2, which is causing the ongoing COVID-19 pandemic that has resulted in 890,392 deaths from about 27 million cases of infection as of 8 September 2020, has provoked worldwide investigations of CoVs. With the aim of developing efficient strategies for controlling virus outbreaks and recurrences of seasonal virus variants, here we overview the structures, diversities, host ranges and host receptors of all IVs and CoVs and critically review current knowledge of receptor binding specificity of spike glycoproteins, which mediates infection, of IVs and of zoonotic, pandemic and seasonal CoVs.

## 1. Introduction

A pandemic respiratory disease is one of the scariest diseases due to its rapid spread among immunologically naïve humans and due to the fact that there is no vaccine against a new virus strain with a zoonotic origin. In the past two decades, there have been several outbreaks of zoonotic origin in human populations including the chikungunya outbreak in Caribbean countries and South America in 2013–2014 [1], Zika outbreak in the Americas in 2015–2016 [2], Ebola outbreak in 2014–2016 in West Africa [3], HPAI and LPAI outbreaks several times in many countries including Egypt, China, Indonesia, Canada and Australia [4,5], SARS-CoV outbreak in 2002–2004 in 26 countries [6,7], and MERS-CoV outbreak since 2012 in 27 countries [8]. However, a pandemic had not occurred until airborne viruses, including a quadruplex influenza A (H1N1) virus of swine origin and SARS-CoV-2 [9], emerged. The new H1N1 virus caused the 2009 influenza pandemic in June 2009 [10]. SARS-CoV-2, which causes COrona VIrus Disease 2019 (COVID-19), became the first recorded coronavirus pandemic on 11 March 2020 [11]. There are various influenza viruses (IVs) and various coronaviruses (CoVs), which are grouped into 4 genera, alpha, beta, gamma and delta IVs/CoVs (Table 1 and Table 2).

Of the three genera of IVs, alpha IVs (IAVs), beta IVs (IBVs) and gamma IVs (ICVs), that infect humans, eight segmented (−)ssRNA-containing IAVs (A/H3N2/68 and A/H1N1/09 variants) and IBVs (B/Yamagata and B/Victoria lineage-like viruses) with hemagglutinin (HA) spikes cause seasonal influenza epidemics that spread rapidly and cause mild to severe or fatal illnesses. Seasonal influenza vaccines and anti-influenza drugs against IAVs and IBVs are available, but seasonal influenza still causes up to 5 million severe illnesses and up to 650,000 deaths each year [12]. Thus, epidemics of IAVs and IBVs are important public health issues [12]. Seven segmented (−)ssRNA-containing ICVs with hemagglutinin-esterase-fusion (HEF) spikes usually cause mild upper respiratory disease but can cause lower respiratory disease in children and severe illness in infants [13]. Other seven segmented (−)ssRNA-containing IDVs with HEF spikes mainly infect cattle and cause respiratory illness [14]. These IDVs seem to have a zoonotic potential to infect humans, but whether they can cause illness in humans remains unknown [15]. Among all known influenza viruses, only IAVs are subtyped according to their HAs (H) and neuraminidase (NA or N) spikes into H1–H18 and N1–N11. Several subtypes of IAVs, including H1N1, H3N2 and H5N1, have crossed the species barrier to infect a variety of birds and mammals including humans, indicating that they have zoonotic potential. IAVs not only mutate quickly but also prefer to reassort with other IAVs to form a new strain. Due to these properties, IAVs have caused four pandemics in the past 102 years [10,16]. These viruses continued to threat human health seasonally. However, the first recorded pandemic virus, A/H1N1/1918-derived virus, disappeared from human circulation after the appearance of the A/H2N2/1957 pandemic virus containing five gene segments (PB2, PA, NP, M and NS) from human A/H1N1/1918-derived virus. Similarly, A/H2N2/1957-derived virus disappeared after the appearance of A/H3N2/1968 pandemic virus containing six gene segments (PB2, PA, NP, NA, M and NS) from human A/H2N2/1957-derived virus. In 1977, A/H1N1/1918-derived virus reemerged, probably due to accidental release from a laboratory, as a low-grade A/H1N1/1977 pandemic virus that primarily affected young immunologically naïve people [17,18] who were born after the end of the H1 period (1957 and later). This resulted in two subtypes of IAVs circulating in humans, A/H1N1/1977-derived and A/H3N2/1968-derived viruses. After the appearance of the A/H1N1/2009 pandemic virus containing one human gene segment (PB1) from A/H3N2/1968-derived virus and three gene segments (H1, NP and NS) from classical swine A/H1N1 virus believed to have been transmitted from A/H1N1/1918-derived virus between 1918 and 1920, A/H1N1/1977-derived virus disappeared from human circulation [4,19]. The disappearance of that virus resulted in only A/H1N1/2009-derived and A/H3N2/1968-derived viruses remaining in circulation in humans. Thus, IAVs are important not only because human IAVs cause seasonal influenza but also because nonhuman IAVs cause farm animal diseases, sporadic zoonotic outbreaks and periodic unpredictable pandemics. 

Of the four genera of single linear (+)ssRNA-containing CoVs, only αCoV members (HCoV-229E and HCoV-NL63) and βCoV members (HCoV-OC43, HCoV-HKU1, SARS-CoV, MERS-CoV and SARS-CoV-2) have so far been reported to infect humans. HCoV-229E, HCoV-NL63, HCoV-OC43 and HCoV-HKU1 circulating in humans usually cause mild upper respiratory diseases [20], and there is still no vaccine or antiviral drug against these HCoVs [21]. Zoonotic SARS-CoV, a highly lethal CoV causing SARS disease, emerged in 2002. However, no SARS cases were reported after 29 April 2004 and the World Health Organization (WHO) officially announced on 18 May 2004 that the SARS outbreak had been contained [22]. Zoonotic MERS-CoV, another highly lethal CoV causing MERS disease, emerged in 2012. MERS cases are still being reported to WHO [8]. An anti-MERS drug and MERS vaccine are still not available. Zoonotic SARS-CoV-2 emerged in December 2019 and is causing the ongoing COVID-19 pandemic [11]. Many efforts are being made to develop COVID vaccines and anti-COVID drugs. As in the case of IAVs, CoVs have been isolated from a wide range of host species, and some of the isolated CoVs are zoonotic with the potential to cause an unpredictable pandemic. CoVs must therefore be controlled (Table 2). 

The structures, diversities, host ranges and receptors of IVs and CoVs are summarized in this review. The host range of an infection depends on specific interactions between the virus strain and the host species. We will critically review current knowledge of receptor binding specificity, a crucial determinant of the host range, of IVs and human-infecting CoVs with appropriate viral historical perspective.

## 2. Overview of Structures, Host Ranges and Receptors of Influenza Viruses and Coronaviruses

**Figure 1:**
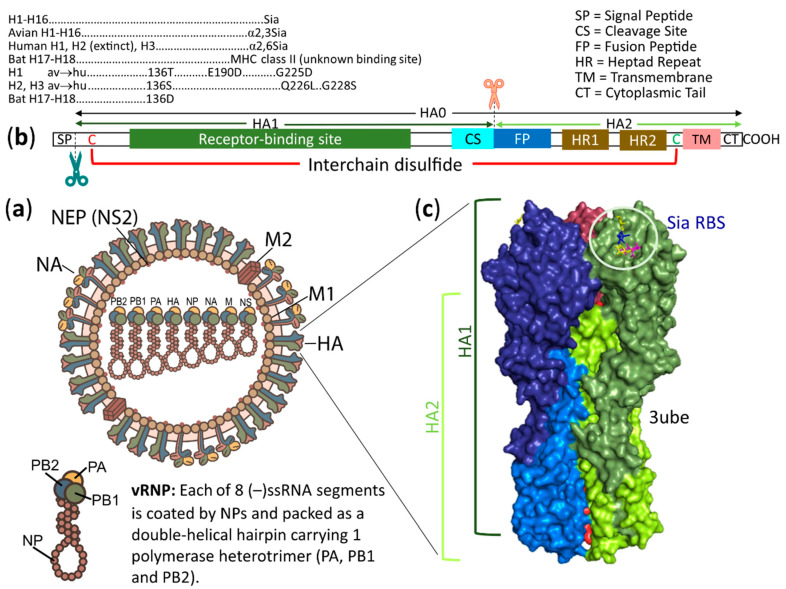
Schematic structures of an IAV and its HA. (**a**) The virion is pleomorphic from spherical to filamentous. It contains eight negative sense, single-stranded RNA genomic segments, each coated with nucleoproteins (NPs) and bound to a heterotrimeric RNA polymerase complex of PA, PB1 and PB2 that forms as a viral ribonucleoprotein (vRNP). All 8 vRNPs are surrounded by a lipid bilayer (envelope). Whereas the inner layer of the envelope is attached to matrix 1 (M1) proteins bound to vRNPs and to nuclear export proteins (NEPs, formerly named NS2 proteins), the outer layer is spiked with HAs and NAs and channeled with matrix 2 (M2) proteins. (**b**) Diagram of an unfolded HA polypeptide. The signal peptide (SP) at the N-terminus is removed during the cotranslational translocation of a nascent HA into the ER [134]. The synthesized single polypeptide HA is designed to be HA0 (inactive form) that carries HA1 and HA2 subunits. The HA1 subunit, in addition to being a major antigen, contains a receptor binding site, which binds specifically to a sialyl glycan as indicated, except for H17 and H18 viruses. A cleavage site (CS) is located at the end of the HA1 subunit. The HA1 CS usually contains a single basic aa (X-R/K) that can be cleaved extracellularly by tissue-specific expressed trypsin-like serine endoproteases. In contrast, the HA1 CS with additional basic amino acids (R/K-R) may be cleaved intracellularly by ubiquitous proteases (furin and PC5/6) in the trans-Golgi compartment recognizing the R-X-R/K-R motif or cleaved extracellularly by ubiquitous proteases (MSPL and TMPRSS13) in the plasma membrane recognizing the R/K-X-R/K-R motif, and the virus can thus cause systemic infection and is classified as a highly pathogenic (HP) virus [135,136]. After the cleavage, the HA1 and HA2 subunits remain disulfide-linked between Cys (C, red) and Cys (C, green) of the two subunits, giving the fusion-active form of the HA. The HA2 subunit carries a hydrophobic fusion peptide (FP) at the N-terminus followed by heptad repeats 1 and 2 (HR1 and HR2). Upon activation at low pH, these 3 regions work cooperatively to fuse viral and cellular membranes together, releasing vRNPs into the host cell. The HA2 subunit also contains a transmembrane domain (TM) anchoring the viral membrane (envelope) and promoting full fusion [137]. A hydrophilic cytoplasmic tail (CT) at the C-terminus of the HA2 subunit can also affect the fusion process [138]. (**c**) Side view of a surface diagram of a trimeric HA. This representative viral HA is from pdb ID of 3ube, which showed a 2009 pandemic HA in complex with lactoseries tetrasaccharide c (LSTc, Neu5Acα2,6Galβ1,4GlcNAcβ1,3Galβ1,4Glc) [139]. Each monomer of the HA trimer is colored as follows: deep blue, HA1 and marine blue, HA2; green smudge, HA1 and lemon, HA2; raspberry, HA1 and red, HA2. The receptor binding site, which carries a part of the LSTc ligand (magenta stick, Neu5Ac; yellow stick, Gal; blue stick, GlcNAc; yellow stick, Gal), is located on each globular head, which is a part of HA1 on a stalk composed of the remaining part of HA1 and all HA2.

**Figure 2:**
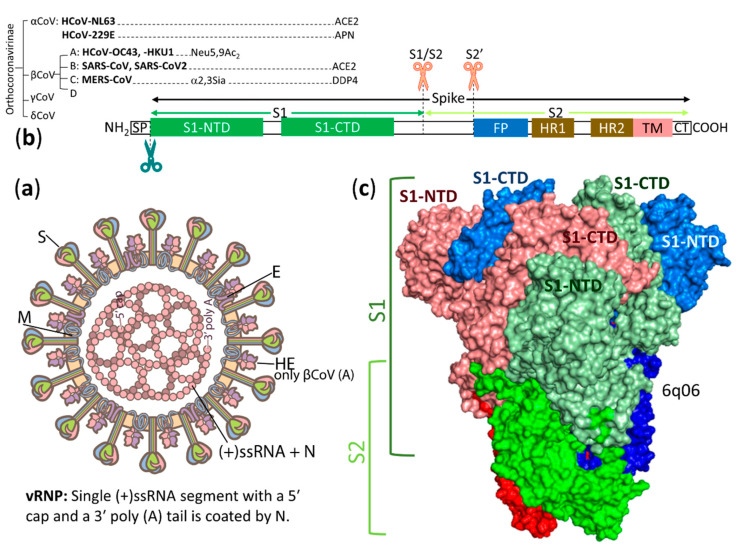
Schematic structures of a CoV and its spike protein. (**a**) The CoV is a pleomorphic spherical enveloped particle. It contains a linear positive-sense, single-stranded RNA with a 5′ cap and a 3′ poly(A) tail that are enclosed by nucleocapsid (N) proteins. The lipid bilayer envelope carries 3–4 structural proteins. (i) Membrane (M) proteins are the most abundant small triple-spanning transmembrane proteins that define the virion morphology and are major coordinators of virion assembly [140]. (ii) Envelope (E) proteins are minor channel-spanning transmembrane proteins that work together with the M proteins for virion assembly, budding and release [141]. (iii) Spike (S) proteins are homotrimeric type I transmembrane glycoproteins [142] protruding from the virion envelope (20 nm in length) that resemble a crown (Latin: corona) under an electron microscope. S proteins are major antigenic surface proteins and are critical for virion entry into a specific host cell by binding to a specific receptor(s) on the host cell surface and mediating membrane fusion [143]. (iv) The members of βCoV lineage A have additional short hemagglutinin esterase (HE) spikes (5 nm in length), which are homodimeric type I transmembrane glycoproteins. CoV HE has the potential to evolve its *O*-Ac-Sia receptor-binding specificity and activity, along with its companion S proteins, in balance with its receptor-destroying sialate *O*-acetylesterase activity for efficient virus infection and spread [86,94,144]. (**b**) Diagram of the unfolded S polypeptide composed of an N-terminal signal peptide (SP), an S1 subunit and an S2 subunit. The N-terminal signal peptide is a short type 1 signal hydrophobic peptide probably cleaved during cotranslational transport across the endoplasmic reticulum [145]. The S1 subunit carries 2 important domains, an S1 N-terminal domain (S1-NTD or S1^A^) and an S1 C-terminal domain (S1-CTD or S1^B^), one of which or both of which function as a host receptor-binding domain depending on the virus. Host receptors of CoVs affecting humans are shown above the bound S1 domain. The S1/S2 boundary with multiple basic aa residues may be potentially cleaved by furin during virus exit, while the monobasic cleavage site may be cleaved by a target cell protease, such as TMPRSS2 (trypsin-like protease) and cathepsin L [146], generating the S1 and S2 subunits with a noncovalently link [147]. The S2 subunit carries 5 regions. (i) The S2′ cleavage site may contain monobasic or two basic residues that must be cleaved by a host protease [146]. Both S1/S2 and S2′ cleavage sites must be cleaved to enable its S protein to mediate membrane fusion. (ii) A fusion peptide (FP) that mediates fusion of the virion envelope with the cellular plasma membrane or with the cellular endosomal membrane, followed by (iii) 2 heptad repeats (HR1 and HR2) promoting fusion [147], (iv) a transmembrane (TM) domain anchoring to the envelope and (v) a cytoplasmic tail (CT). (**c**) Side view of a surface diagram of a trimeric CoV S protein (pdb: 6q06 [148]). Each monomer of the S trimer is colored as follows: salmon, S1 and red, S2; marine blue, S1 and blue, S2; pale green, S1 and green, S2. S1-NTD and S1-CTD are on each S1 head above each S2 stalk.

Table 1 and Table 2 show the host ranges, diseases, receptor-binding viral spikes and receptors of influenza viruses (IVs) and coronaviruses (CoVs), respectively. According to the International Committee on Taxonomy of Viruses [117], IVs in the family *Orthomyxoviridae* are divided into four genera: *Alphainfluenzavirus*, *Betainfluenzavirus*, *Gammainfluenzavirus* and *Deltainfluenzavirus* (previously, *Influenzavirus A*, *B*, *C* and *D*, respectively). CoVs in the subfamily *Orthocoronavirinae* of the family *Coronaviridae* are divided into four genera: *Alphacoronavirus* (αCoV, previously group 1), *Betacoronavirus* (βCoV, previously group 2), *Gammacoronavirus* (γCoV, previously group 3) and *Deltacoronavirus* (δCoV, a new group). The genus βCoV is subdivided into four lineages: A, B, C and D. Both IVs and CoVs are enveloped single-stranded RNA viruses. However, IV genomes are negative-sense, linear RNA segments (eight segments for influenza A and B viruses and seven segments for influenza C and D viruses [86]), whereas the CoV genome is a positive-sense, linear RNA molecule [118]. Various CoVs cause a variety of diseases in mammals and birds [118]. In contrast, IVs in mammals (except for bat H17 and H18 viruses that were found in rectal swabs [119,120]) mainly cause respiratory infection [4,42], whereas infection of IVs in birds occurs in the intestinal and respiratory tracts [121,122]. The virus host range is chiefly determined by the viral spike glycoproteins carrying receptor binding sites for attachment of host receptors initiating virus infection. To bind to host receptors, influenza A viruses (IAVs) and influenza B viruses (IBVs) use hemagglutinin (HA), while influenza C viruses (ICVs) and influenza D viruses (IDVs) use hemagglutinin-esterase-fusion (HEF) to bind to host receptors as indicated in Table 1. Only IAVs have a broad range of host species. An IAV structure with HA spikes and the position of a receptor binding site (RBS) on an HA1 subunit are shown in Figure 1. Avian IAVs from wild birds are thought to be the origin of all mammalian IAVs (not known for bat H17 and H18 viruses) including all human IAVs [123]. CoVs use their spike (S) glycoproteins in N- and/or C-terminal domains of the S1 subunit, S1-NTD and/or S1-CTD, respectively, as indicated in Table 2. Specially, CoVs in lineage A of the genus βCoV have additional spike glycoproteins, hemagglutinin-esterases (HEs), possibly acquired from influenza C virus (ICV) during mixed infection [124]. A CoV structure with S and HE spikes and positions of S1-NTD and S1-CTD on the S spike are shown in Figure 2. Bat CoVs are likely to be the ancestral origins of two seasonal αCoVs, HCoV-229E [125,126] and HCoV-NL63 [127], two zoonotic lineage B and lineage C βCoVs, SARS-CoV (which has disappeared in humans) [128,129,130] and MERS-CoV [108,131], respectively, and an ongoing pandemic lineage B βCoV, SARS-CoV-2 [132,133]. Rodent CoVs are likely to be the ancestral origins of two seasonal lineage A βCoVs, HCoV-OC43 and HCoV-HKU1 [85]. So far, γCoVs and δCoVs have been mainly found in birds [85]. This review will focus on human viruses and potential zoonotic viruses and the main spike glycoproteins determining host-specific virus infection.

## 3. Sialyl Glycan Receptor-Dependent Recognition of Influenza A (H1–H16) Viruses, Egyptian Fruit Bat-Isolated Influenza A Virus, Influenza B, C and D Viruses and Lineage A βCoVs (β1CoVs)

### 3.1. Influenza A (H1–H16) Viruses Use Siaα2,3/2,6Gal Receptors

**Figure 3:**
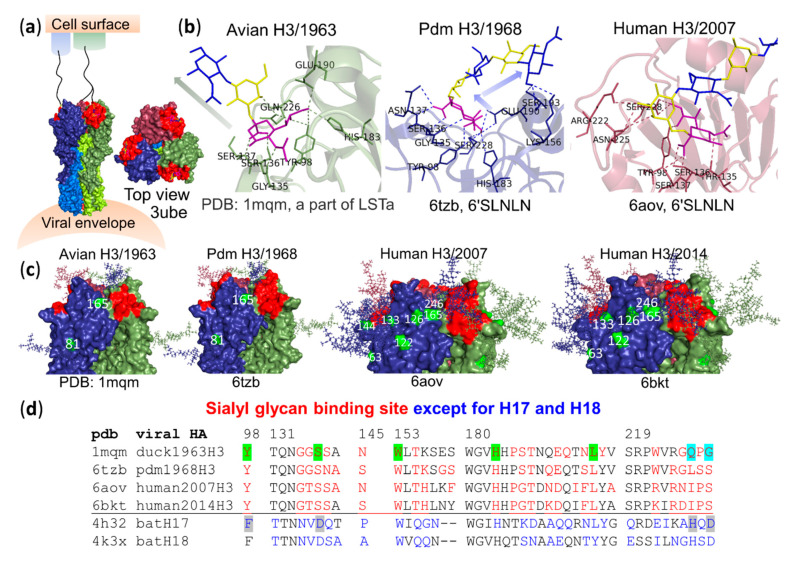
Binding of avian, pandemic (pdm) and seasonal HAs to specific sialyl glycans. (**a**) HA trimer binding to sialyl glycan receptors on the host cell surface. (**b**) Direct interactions in stick models between aa residues in avian/1963 (green smudge color), pdm/1968 (deep blue color) and seasonal/2007 (raspberry color) H3 HA receptor binding sites and sugar residues, a part of lactoseries tetrasaccharide a (LSTa; Neu5Acα2,3Galβ1,3GlcNAcβ1,3Galβ1,4Glc), 6′sialyl di-*N*-acetyllactosamine (6′SLNLN; Neu5Acα2,6(Galβ1,4GlcNAc)_2_) and 6′SLNLN, respectively. Neu5Ac, magenta; Gal, yellow; GlcNAc, blue. (**c**) Comparison of the number of glycosylation sites (green residues) on the HA1 globular head trimer (surface diagram) between avian, pdm and seasonal H3 HAs. Glycans were *in silico* added into *N*-glycosylation sites (NXS/T, X = any residue except for Pro) on the H3 HA heads by GlyProt and are shown as sticks with organic spheres. (**d**) Alignment showing aa changes in RBS (H3 numbering). Amino acids in avian, pdm and seasonal H3 HAs and in H17–H18 HAs generating a shallow pocket are labeled red and blue, respectively. Highly conserved amino acids giving direct interactions with Sia are highlighted in green. Well-known amino acids in HA sequences that are critical for determination of the sialyl linkage-type of avian and human host cells are highlighted in cyan. 1mqm, 6tzb, 6aov and 6bkt are from [149,150,151,152], respectively. The amino acid alignments shown in this figure and in the other figures were performed by using Clustal Omega [153].

HAs of H1–H16 viruses recognize specific sialyl glycans on the host epithelial cell surface, as a crucial step mediating virus infection (Figure 3a). IAVs from avians, either wild birds or domestic birds, typically prefer the α2,3Sia terminal. Surprisingly, a recent study showed that some gull/tern H16 viruses prefer α2,6Neu5Ac over or equal to the α2,3Neu5Ac terminal of synthetic sialylglycopolymers [23]. It was suggested that the particularly distinctive receptor-binding specificity of H16 viruses may be related to their HAs containing A138S (found in human 1977-derived H1N1 viruses, reducing binding to α2,3Neu5Ac receptors) and E190T (the amino acid (aa) at position 190 determining binding specificity of H1N1 viruses to the sialyl linkage type) [23]. Usually, viruses adapt to bind to sialyl glycans dominant in the host target tissues. Information on virus collection from oral, nasal, nasopharyngeal, cloacal or feces swabs and sialyl glycan analysis of tissues of specific wild birds shedding the virus in the sample collection may lead to a better understanding of why some H16 viruses display binding distinct from that of other avian viruses. Binding preference for the internal part of sialyl glycans appears to differ among different viruses based on birds of isolation as indicated in Table 1. 

Several zoonotic influenza virus subtypes (Table 1) including avian subtypes H5N1, H7N9 and H9N2 and swine subtypes H1N1, H1N2 and H3N2 have been occasionally reported to cross the species barrier to infect humans [57]. However, human-to-human transmission of nonhuman viruses has been limited and non-sustained [154]. Viruses in four historical pandemics acquired strong binding to human-type α2,6Neu5Ac receptors for efficient human-to-human transmission [19,155,156,157]. (i) The H1N1 Spanish pandemic in 1918–1919 was found to include at least two strains with distinct receptor-binding properties during the pandemic period [155]. First, viral HAs have a single aa substitution, E190D, in the receptor-binding site (RBS) and bind to both avian-type and human-type receptors. Second, there are two aa substitutions in the HA RBS, E190D and G225D, that enable HA adaptation to bind only to α2,6Neu5Ac receptors. (ii) The H2N2 Asian pandemic in 1957–1958 had virus isolates from two stages of the pandemic. In the early pandemic stage, virus isolates can be divided into three subpopulations based on receptor binding specificities: avian-like viruses with 226Q and 228G in the HA RBS, atypical viruses with Q226L and 228G, and classic human viruses with Q226L and G228S that have preferential binding to avian-type receptors, both avian-type and human-type receptors, and human-type receptors, respectively. In the subsequent stage, all virus isolates have Q226L and G228S substitutions with preferential binding to human-type receptors [156]. (iii) The virus in the H3N2 Hong Kong pandemic in 1968–1969 had the same acquisition of Q226L and G228S substitutions in the HA RBS as that in the H2 pandemic for switching from avian-type to human-type receptor binding preference [157]. (iv) The virus in the H1N1 swine pandemic in 2009–2010 had 190D and 225D in the HA RBS as in the swine H1 HA RBS recognizing human-type α2,6Neu5Ac receptors that are abundant in the porcine lung, which is the main site of swine IAV replication [19,39]. Protein engineering by chimeragenesis and site-directed mutagenesis of H1 proteins suggested that A200T and A227E substitutions in the H1 swine pandemic were responsible for efficient and sustained human-to-human transmission. Molecular modeling revealed hydrogen bond formation between T200 and Q191 in the 190-helix that is important for receptor binding preference of H1 HAs and between E227 and Gal next to Sia [158]. 

Based on historical data, after a pandemic virus continued to circulate as a seasonal strain, the preexisting seasonal virus, which donated at least three gene segments to the pandemic virus, disappeared from human circulation. The disappearance of 1918-derived H1N1, 1957-derived H2N2 and 1977-derived H1N1 (being the 1918-derived H1N1, recurrent from a research laboratory in 1977, same as the classical swine H1N1 viruses) [159] has resulted in only 1968-derived H3N2 and 2009-derived H1N1 viruses remaining in human circulation. Despite binding to human-type receptors being essential for influenza virus transmission among humans, the binding of 1968-derived H3N2 viruses to the human-type receptor analog α2,6 sialyl *N*-acetyllactosamine (6′SLN)-polyacrylamide started to decrease significantly in 2001 and seemed to be completely lost in 2010 [160]. However, after the discovery [161] and the widespread use of long α2,6 sialylated *N*-glycans with multiple LN repeats for studies on influenza virus binding specificity, it appeared that 1968-derived H3N2 viruses have evolved binding preference for human-type receptors with LacNAc (LN) repeats [60,162]. Based on the binding preferences to short 3′SLN and 6′SLN and long 3′SLNLNLN and 6′SLNLNLN linked to a polyglutamic acid, IAVs can be divided into two groups [19]. Group 1 are avian viruses, including H5N1 and H5N3 viruses, that preferentially bind to terminal α2,3Neu5Ac with either short or long LN chains. Group 2 consists of viruses that preferentially bind to terminal α2,6Neu5Ac, and the viruses can be further divided into two subgroups. Subgroup 2-1 includes swine H1N2/2008 and pdm H1N1/2009 viruses, which can bind to both short and long α2,6 sialylated glycans. These results support the hypothesis that pigs are vessels to generate viral HAs with pandemic potential [41]. However, the pdm H1N1/2009 viruses acquired at least two amino acids that are different from the swine H1 HA, A200T and A227E, and they are responsible for the binding differences in fetuin, chicken erythrocytes and human erythrocytes and are believed to be determinants of the shift in binding specificity from swine-type to human-type [158]. Further investigation to find the α2,6 sialyl glycan structure that is able to clearly distinguish binding specificity between swine and pandemic viruses is needed since such sialyl glycans could be useful for surveillance and prevention of a pandemic. Subgroup 2-2 consists of long-term circulating human viruses including human H3N2/2008 viruses and human H1N1/2004 and H1N1/2006 viruses, which have binding preference to the long α2,6 sialyl glycan. 

Structural comparison of avian-type and human-type receptors interacting with the receptor binding sites of avian H3/1963, pandemic (pdm) H3/1968 and human H3/2007 (Figure 3b) revealed that trisaccharide Neu5Acα2,3Galβ1,3GlcNAc of lactoseries tetrasaccharide a (LSTa) interacts with the avian H3/1963 binding site in a cone-like topology (1mqm [149]), whereas 6′SLNLN interacts with pandemic H3/1968 (6tzb [150]) and human H3/2007 binding sites in an umbrella-like topology (6aov [151]). Residues 226 and 228 are important in determining sialyl linkage binding specificity. As shown in Figure 3b, Q226 in the avian H3/1963 binding site directly forms hydrogen bonds with Sia-1 and Gal-2 of LSTa, whereas S228 in the pdm H3/1968 binding site directly forms a hydrogen bond with Sia-1 of 6′SLNLN and S228 in the human H3/2007 binding site directly forms two hydrogen bonds with Sia-1 of 6′SLNLN. Although no direct interaction of residue 226 in pdm and human H3 HAs was found, a previous study suggested that L226Q mutation in the HA decreases α2,6Neu5Ac binding preference. Both G228 and S228 can be found in H3 avian HAs, whereas only S228 is found in human H3 HAs [149]. L226 is not conserved during circulation in humans; L226V and V226I substitutions were observed before 2001 and in 2004, respectively [160]. 

Similar to the H1 HA receptor binding site [10], two sets of human receptor binding residues provide networks to make contact with the long human-type receptor that results in an umbrella-like topology of the receptor. (i) A base region Neu5Acα2,6Galβ1- motif is governed by residues 131–138 in a 130-loop, residues 140–145 in a 140-loop and residues 219–228 in a 220-loop [163]. Figure 3b shows direct H-bond formation between Y98, G135, S136, N137, H183, E190 and S228 in the pdm H3/1968 binding site and Sia-1 of 6′SLNLN. In the human H3/2007 binding site, Y98, T135, S136, S137, S228, R222, and N225 make direct H-bonds with Sia-1 and Gal-2, respectively, of 6′SLNLN. (ii) The extension region -4GlcNAcβ1,4Galβ1,4GlcNAc motif is governed by residues 190–196 in a 190-helix and residues 156–160 in a 150-loop [163], and S193 and K156 in the pdm H3/1968 binding site were observed to generate direct H-bonds with GlcNAc-5 of 6′SLNLN. Amino acid change in HA during co-evolution with humans occurs to evade human immunity. Not only is there a change in antigenicity but the number of glycosylation sites masking antigenicity also increases over time as shown in Figure 3c; the numbers of glycosylation sites/monomer are two for avian H3/1968 HA, two for pdm H3/1968 HA, seven for human H3/2007 HA and six for human H3/2014. The limitation of increase in the number of glycosylation sites might be because the change of the virus must have a balance between mutation and selection for optimal immune evasion and infection. Taken together, the change in receptor binding specificity of long-term circulating human IAVs from short and long to long α2,6 sialylated glycans may have resulted from aa change in the RBS (Figure 3b,d) and an increase in glycosylation sites surrounding the RBS, possibly making the shallow RBS deeper (Figure 3c). The differences in receptor binding preferences of avian, pandemic and long-term circulating human IAVs are associated with viral pathology along the human respiratory tract containing different sialylated glycan structures. The preferential binding of avian and pandemic viruses to both short and extended receptors can typically cause diffuse alveolar damage, resulting in greater severity than that caused by long-term circulating human viruses with preference for long receptors that rarely infect human alveoli [164,165]. This correlates well with our finding that human alveolar *N*-glycans consist of mainly short receptors, 22.32: 0.17: 16.10: 0.15 mol% (Neu5Acα2,3LN: Neu5Acα2,3(α1,3fucosylated LN): Neu5Acα2,6LN: Neu5Ac-LN-LN), of total human alveolar *N*-glycans [19]. Sialyl *N*-glycans with various numbers of LN units (up to 10 units) have been reported in human lungs (principally terminated in α2,3Neu5Ac [166]) and the human bronchus, whereas fewer extended LN profiles can be detected in the human nasopharynx [167]. Although structures of glycans in the human trachea have not be determined, the pdm H1N1/2009 virus was found at higher levels in tracheal aspirate specimens than in throat or nasopharyngeal swabs [168]. Uncomplicated long-term circulating human viruses are related to tracheobronchitis [169].

### 3.2. Egyptian Fruit Bat-Isolated Influenza A Virus Uses Siaα2,3Gal Receptors

In 2019, Kandeil et al. [64] reported a new IAV isolated in 2017 from Egyptian fruit bats (*Rousettus aegyptiacus*, family *Pteropodidae*) in an abandoned mudbrick house in a densely inhabited agricultural village in the Nile Delta, Egypt. The new IAV was found more frequently in oral swabs than in rectal swabs. Each of eight genomic segments of this newly characterized bat influenza A/bat/Egypt/381OP/2017 virus was shown to have nucleotide (nt) and aa sequences similar to those in genes of other avian IAVs isolated from wild birds, except for those in the PA gene, which are similar to those in the PA gene of an equine IAV (H7N7). The HA protein of the Egyptian bat IAVs is closely related to the group 1 cluster of HA subtypes with highest similarity (73% identity) to the H9 HA of influenza A/mallard/Ohio/13OS3856/2013 virus (H9N2). A receptor binding assay indicated that the Egyptian bat virus possessing Q226 (H3 numbering) in the RBS showed a clear binding preference for α2,3sialyllactose receptors over α2,6sialyllactose receptors, suggesting that Siaα2,3Gal receptors might be abundant in the infection sites in Egyptian fruit bats. Further investigation of that possibility is required. The virus was speculated to originate from an avian host, and that speculation was supported by the finding that the virus can grow well in allantoic fluid cavities of embryonated chicken eggs. The virus can also propagate in MDCK cells and in the lungs of C57BL/6 mice and BALB/c mice, indicating the possibility of the virus causing infection in other mammalian species. Thus, surveillance of IAVs among bats and distribution in other animals should be performed.

### 3.3. Influenza B Viruses Use Siaα2,3/2,6Gal Receptors

In 1940, a new serotype of influenza viruses was isolated and designated type B. The first strain was named B/Lee/1940. Influenza B viruses have continued to cause respiratory disease in humans with antigenic change. Although HA and NA antigenic differences within influenza B viruses (IBVs) are not sufficient to separate antigenic subtypes, there were sufficient antigenic differences to classify IBVs into two lineages: (i) Victoria lineage B/Victoria/2/1987-like and (ii) Yamagata lineage B/Yamagata/16/1988-like viruses [170]. Consequently, morbidity and mortality-associated seasonal influenza is currently caused by the two lineages of IBVs and two subtypes of IAVs, 1968-derived H3N2 and 2009-derived H1N1 viruses. In contrast to IAVs, IBVs infect mainly humans, although there are sporadic reports of IBV infection in seals, pigs, horses, pheasants and dogs [65]. Similar to IAVs, IBVs have eight (-)ssRNA genome segments and possess receptor-binding and -destroying activities on different molecules, homo-trimeric HA and homo-tetrameric NA glycoproteins. Clinically approved NA inhibitors (NAIs), including zanamivir, oseltamivir, laninamivir and peramivir, are now used for treatment of infection with not only IAVs but also IBVs [171]. Several chemical compounds that have been developed as anti-influenza A NAs, including Neu5Ac2en mimetics for minimizing side effects on human Neu1-Neu4 enzymes [172,173] and NA covalent inhibitors for irreversible NA inhibition [174] and *Psidium guajava* Linn. (guava) tea [175] and povidone-iodine that possess anti-influenza A sialidase activities [176] might be able to inhibit influenza B viruses.

Receptor binding specificity determines the site of virus infection. It appears that wild-type influenza B/Victoria HAs possessing G141, R162 and D196 [67] and B/Yamagata HAs with F95 and N194 [68] clearly exhibit binding preference to human-type α2,6Neu5Ac receptors. Investigation of receptor binding preference of IBV clinical isolates in Taiwan during the period from 2001 to 2007 (Table 1) revealed that (i) 83% of Yamagata-like strains prefer α2,6Sia receptors, whereas 17% of them prefer both α2,3Sia and α2,6Sia and (ii) 54% of Victoria-like strains prefer both α2,3Sia and α2,6Sia, whereas 25% of them prefer sulfated glycan, either β-Gal-3-sulfate or 6-HSO_3_-Galβ1,4GlcNAc, and 21% of them prefer α2,6Sia. The viruses with dual α2,3Sia and α2,6Sia-binding preferences were shown to be associated with bronchopneumonia and gastrointestinal symptoms [66]. These findings indicate that the evolution of receptor binding specificity in IBVs in circulation is different from that in IAVs and indicate tissue tropism and pathogenicity of IBVs, possibly affecting virus transmission.

### 3.4. Influenza C Viruses Use Neu5,9Ac_2_

In 1947, a new influenza virus without cross-reactive antisera against IAV (PR8) and IBV (Lee) was first isolated by R.M. Taylor from throat washings of a New York man during an influenza outbreak [177]. It was later designated type C and the first strain was named C/Taylor/1233/1947. ICV usually causes mild upper respiratory infection but can cause lower respiratory infection in children less than 2 years of age [178]. Most humans acquire antibodies to ICV at a young age [178,179] and antigenicity of ICV is stable, with no antigenic change being detected for at least 30 years [71]. These facts may be related to the limited outbreaks of ICV in humans, mainly in children. Although ICV antigenicity is stable, comparison of HE gene sequences in viruses isolated from 1947 to 2014 demonstrated that there are six lineages comprised of C/Taylor/1233/1947, C/Kanagawa/1/1976, C/Mississippi/1980, C/Aichi/1/1981, C/Yamagata/26/1981 and C/Sao Paulo/378/1982 [71]. ICVs have also been isolated from pigs [69] and cattle [70] (Table 1).

Different from IAVs and IBVs, ICV possesses hemagglutinin and receptor-destroying enzyme (RDE) on the same homotrimeric glycoprotein having multifunctional hemagglutinin (receptor-binding and membrane fusion activities) and esterase (receptor-destroying activity) and so-called hemagglutinin-esterase-fusion (HEF) protein [180]. The glycoprotein HEF spikes are encoded by the fourth gene segment, and only the ICV (-)ssRNA genome is comprised of only seven gene segments [181]. 

Thin-layer chromatography (TLC), gas-liquid chromatography (GLC) and high-performance liquid chromatography (HPLC) analyses of rat alpha 1-macroglobulin (RMG) and bovine submaxillary mucin (BSM) incubated with ICV in comparison with those incubated with neuraminidase from *A. ureafaciens* revealed that RMG and BSM incubated with ICV have a reduced amount of Neu5,9Ac_2_ but an increased amount of Neu5Ac. After confirmation by using purified Neu5,9Ac_2_ instead of RMG and BSM, it was concluded that RDE of ICV is neuraminate *O*-acetylesterase (9-*O*-acetyl *N*-acetylneuraminate *O*-acetylhydrolase (EC 3.1.1.53) catalyzing removal of the 9-*O*-acetyl group from Neu5,9Ac_2_, not cleaving the terminal Neu5Ac from glycoconjugate [182]. RMG and BSM can potentially inhibit hemagglutination by ICV at 4^o^C, and their inhibitory effects were abolished by pre-incubation of RMG and BSM with ICV at 37^o^C [182]. This evidence suggested that Neu5,9Ac_2_ is a receptor of ICV on the cell surface.

Receptor binding analysis of C/Johannesburg/1/66 classified in C/Aichi lineage [71] on a sialoglycan microarray showed that the virus predominantly binds to Neu5,9Ac_2_α2,6Galβ1,4GlcNAc β1,2Manα3(Neu5,9Ac_2_α2,6Galβ1,4GlcNAcβ1,2Manα6)Manβ1,4GlcNAcβ1,4GlcNAcitol-AEAB [72]. Further studies by using ICVs from other lineages may help to clarify whether receptor binding specificity of all ICVs to Neu5,9Ac_2_ depends on the α2,6 linkage or not.

### 3.5. Influenza D Viruses Use Neu5,9Ac_2_ and Neu5Gc9Ac Receptors

In 2011, a novel virus isolated from a nasal swab of a 15-week-old pig with influenza-like symptoms in Oklahoma in the USA was found to possess seven (-)ssRNA genomic segments and HEF spike glycoproteins and to share approximately 50% overall aa sequence identity with human ICVs, and it was named C/swine/Oklahoma/1334/2011 (C/OK) [183]. At first, it was suggested to be a new subtype of ICVs due to (i) no cross-reaction of C/OK with human ICVs determined by hemagglutination inhibition assays and (ii) a wider cellular tropism of C/OK than that of a human ICV determined by cell culture studies [183]. In 2016, however, it was determined by the International Committee on Taxonomy of Viruses that this novel influenza virus is distinct from other types, and it was officially classified in a new genus, *Deltainfluenzavirus*, and so-called influenza D virus (IDV, type (species) D). As shown in Table 1, in addition to pigs, IDVs have been isolated from cattle and have so far been classified into three lineages: D/OK (D/swine/Oklahoma/1334/2011-like viruses), D/660 (D/bovine/Oklahoma/660/2013-like viruses) and D/Japanese, with D/Japanese lineage being further classified into 2 sublineages, D/Yama2016 (D/bovine/Yamagata/10710/2016-like viruses) and D/Yama2019 (D/bovine/Yamagata/1/2019-like viruses), based on phylogenetic and antigenic analyses [73]. Although there has been only serological evidence suggesting that IDV can infect humans [15], the virus may acquire mutations to potentially infect humans and to cause influenza illness in humans. 

The host range of IVs is primarily determined by receptor binding specificity of the viruses. Recently, Liu et al. compared receptor binding specificities of IDVs and their related ICVs by a sialoglycan microarray approach [72]. Strain D/swine/Oklahoma/1334/2011 (D/OK) showed preferential binding to Neu5,9Ac_2_ and Neu5Gc9Ac either linked to α2,6Gal or α2,3Gal and strain D/bovine/Oklahoma/660/2013 /660) preferred to bind to Neu5,9Ac_2_α2,6Gal, Neu5Gc9Acα2,6Gal and Neu5Gc9Acα2,3Gal, whereas strain C/Johannesburg/1/1966 dominantly recognized Neu5,9Ac_2_α2,6Gal. The broader receptor recognition by IDVs than by human ICV could explain why cellular tropism of IDVs is wider than that of human ICVs. Binding of IDVs to both Neu5,9Ac_2_ and Neu5Gc9Ac, different from human ICV binding to Neu5,9Ac_2_, could be determined by their different HEF-binding pockets. It was shown that different from human ICV HEF of C/Johannesburg/1/1966, swine IDV HEF of D/OK has an open cavity between the 230-helix and 270-loop in the receptor-binding site, which is thought to allow for accommodation of diverse glycan receptors, including Neu5Gc9Ac harboring an extra hydroxyl group on the *N*-acetyl group of C5 Neu5Gc and different sialyl linkages [184]. Further investigation of the structure of the bovine IDV HEF-binding pocket might lead to an understanding of different receptor binding preferences of swine and bovine IDVs. Receptor binding specificity of viruses is believed to be associated with receptors present on the target tissue. Glycoconjugate structures terminated with Neu5,9Ac_2_ and Neu5Gc9Ac along the bovine, porcine and human respiratory tracts have not been determined and further investigation is therefore needed. Previous findings that there is no Neu5Gc production in healthy humans due to mutation of a gene encoding CMP-Neu5Ac hydroxylase, which converts CMP-Neu5Ac to CMP-Neu5Gc [42,185], could explain why human ICVs prefer binding to Neu5,9Ac_2_, whereas swine and bovine IDVs can bind preferentially to both Neu5,9Ac_2_ and 9-*O*-acetylated Neu5Gc.

### 3.6. β1 HCoV-OC43 and β1 HCoV-HKU1 Use Neu5,9Ac_2_ Receptors

HCoV-OC43 strain was first detected in 1967 by an organ culture technique from throat washings of patients with common colds [186], but its complete genomic sequence was not reported until 2004 [187]. HCoV-HKU1 was first characterized in 2005 by Woo et al. at the University of Hong Kong (HKU) from a nasopharyngeal aspirate of a patient with pneumonia [188]. Based on genomic sequences reported so far, there is no bat CoV classified as a βCoV lineage A. Based on phylogenetic analysis, both HCoV-OC43 and HCoV-HKU1 βCoV lineage A probably originated in rodents [85]. While an intermediate host of HCoV-HKU1 remains unknown, HCoV-OC43 is believed to have cattle serving as intermediate hosts from rodents to humans [189].

**Figure 4:**
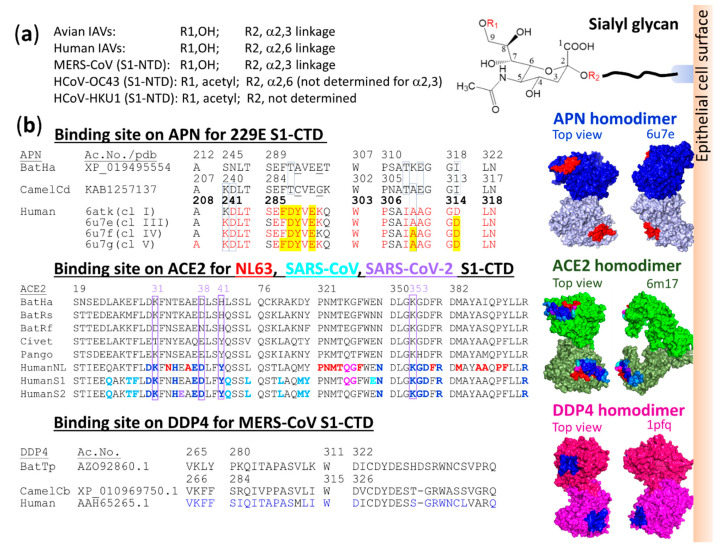
Sialyl glycans and proteins on the epithelial cell surface that are important determinants for IAV and CoV infections. (**a**) Sialyl glycan receptors of IAVs and CoVs. Whereas most IAV HAs prefer the most common Sia, Neu5Ac, over Neu5Gc as a receptor [192], HCoV-OC43 and -HKU1 S1-NTDs recognize Neu5Ac modified with an *O*-acetyl group at the C9-position (R1), Neu5,9Ac_2_ [92,193]. It is well known that sialyl linkage at the C2-position (R2) is a key host determinant of IAVs and that change of their binding preference from α2,3 to α2,6 is a critical factor of avian-to-human host switch [10]. In contrast, there has not been sufficient study on the linkage specificity of HCoV-OC43 and -HKU1 binding, although it is known that HCoV-43 can bind to Neu5,9Ac_2_α2,6Galβ1,4GlcNAc (9-*O*-Ac-6SLN) [193]. Distinct from β1CoVs, additional *O*-acetylation at the C7 or C9-position to Neu5Ac impedes the binding of MERS-CoV (β3CoVs) [112]. Like avian IAV HAs, MERS-CoV S1-NTD prefers Neu5Ac linked to α2,3 over α2,6 [148]. (**b**) Protein receptors of CoVs. Only virus binding site (VBS), receptor amino acids covering the area of the interface between the protein receptor and the indicated viral S1-CTD, are colored. Ac. No., accession number; BatHa, *Hipposideros armiger*; camelCd, *Camelus dromedaries*, batHa, *Hipposideros armiger* (Ac.No., XP_019522954.1); batRs, *Rhinolophus sinicus* (Ac.No., AGZ48803.1); batRf, *Rhinolophus ferrumequinum* (Ac.No., XP_032963186.1); civet, *Paguma larvata* (Ac.No., AAX63775.1); pangolin, *Manis javanica* (Ac.No., XP_017505752.1); human ACE2 (Ac.No., ACT66268); NL, NL63; S1, SARS-CoV; S2, SARS-CoV-2; batTp, *Tylonycteris pachypus*; camelCb, *Camelus bactrianus*. Right: Sialyl glycan structure and top view and side view of surface diagrams of protein receptors. VBSs are colored according to the aa sequences on the left-hand side. 6u7e, 6m17, 1pfq are from [194,195,196], respectively.

HCoV-OC43 does not bind to and agglutinate erythrocytes pretreated with 9-*O*-acetyl esterase from either influenza C virus or bovine CoV [190]. HCoV-HKU1 does not infect primary human ciliated airway epithelial cells pretreated with an expressed HKU1 hemagglutinin-esterase (HE) protein possessing 9-*O*-acetylesterase activity [191]. These findings suggest that both HCoV-OC43 and HCoV-HKU1 bind to 9-*O*-acetylated sialyl glycans (Figure 4a) on the host cell surface for mediating virus infection. As shown in Table 2, the 9-*O*-Ac-Sia receptor-binding function of homodimeric HE proteins, comprised of a receptor-binding (lectin) domain and receptor-destroying domain, of HCoV-OC43 and HCoV-HKU1 was reported to be lost, and its loss was reported to be associated with an accumulation of mutations in the OC43-HE lectin domain or massive deletions found in the HKU1-HE lectin domain during evolution in humans [94]. Binding of the S1 subunit of another type of spike, a homotrimeric spike (S) protein (Figure 2), of HCoV-OC43 and HCoV-HKU1 on human rhabdomyosarcoma cells was shown and was reported to be reduced by pretreating the cells with HKU1-HE, OC43-HE or BCoV-HE, but not by pretreating the cells with MHV-S-HE, possessing 4-*O*-acetylesterase activity [191]. These findings suggested that 9-*O*-Ac-Sia is an essential receptor for infection of HCoV-OC43 and HCoV-HKU1 mediated by the S1 subunit of their S proteins.

**Figure 5:**
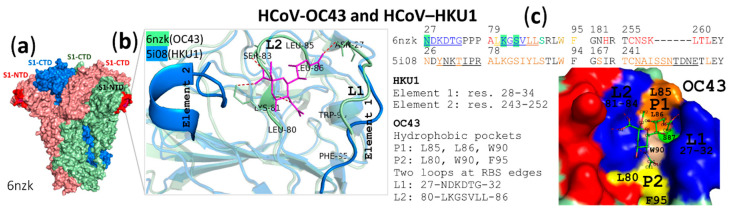
Comparison of Sia binding sites in S1-NTDs of HCoV-OC43 and HCoV-HKU1. (**a**) Side view of the position of RBSs (red) in S1-NTDs of the trimeric HCoV-OC43 spike bound with Neu5,9Ac_2_ in magenta stick (pdb: 6nzk [193]). (**b**) Superimposition between the RBS of HCoV-OC43 pale green spike in complex with Neu5,9Ac_2_ in magenta stick (pdb: 6nzk) and the RBS of HCoV-HKU1 marine spike (pdb: 5i08 [197]). Three aa residues (S83, N27 and K81) that directly interact with Neu5,9Ac_2_ are shown in red sticks. (**c**) Alignment of aa sequences of HCoV-OC43 and HCoV-HKU1 S1-NTDs. Residues directly interacting with the receptor are highlighted green. Right: Residues covering the S1-NTD surface area facing the Neu5,9Ac_2_ receptor, being the RBS, are at the canyon bottom with ridges of HKU1 elements 1 and 2 according to Hulswit et al. [92]. Two loops, L1 and L2, at RBS edges are shown in dark blue and two hydrophobic pockets are shown in orange (P1) and yellow (P2) separated by lightorange (W90) according to Tortorici et al. [193]. Red is the ridge in OC43 S1-NTD corresponding to HKU1 element 1.

The S1 subunit of the S protein is composed of four domains, A through D (S1^A^ through S1^D^) domains from the N-terminus [112]. By using OC43 or HKU1 S1^A^–Fc proteins in a direct binding assay, HCoV-OC43 and HCoV-HKU1 were shown to bind to the receptors via domain A (S1-NTD (Figure 5a), residues 15–302 based on the S protein of OC43 strain ATCC VR-759) [92]. However, binding of HKU1 S1^A^ to its receptors on rat erythrocytes can be detected when HKU1 S1^A^–Fc proteins have been conjugated to nanoparticles but cannot be detected by using free HKU1 S1^A^–Fc proteins (the standard method), indicating the requirement of multivalency of HKU1 S1^A^–Fc proteins for binding to rat erythrocytes. Based on structural analysis, residues 28–34 (element 1) and/or residues 243–252 (element 2) in HKU1 S1^A^ (Figure 5b) were thought to hamper the binding of HKU1 S1^A^. The mutant HKU1 S1^A^ was generated by replacement of one or both of their elements with the corresponding element(s) from bovine coronavirus (BCoV), which is believed to be the ancestor of OC43. The free mutant HKU1 S1^A^–Fc proteins with only one replacement at element 2 were found to bind to rat erythrocytes. The free mutant HKU1 S1^A^–Fc proteins with replacement of both elements showed greater binding to rat erythrocytes. In comparison with binding of the wild-type HKU1 S1^A^ conjugated with nanoparticles to rat erythrocytes, the mutant HKU1 S1^A^ with removal of a glycosylation site at element 2 (N251Q) showed increased binding, and the mutant HKU1 S1^A^ with removal of the glycosylation sites in both elements (N29Q in element 1 + N251Q in element 2) showed greater binding. These findings indicated that binding of HKU1 S1^A^ to its receptors on rat erythrocytes is impeded by both the RBS architecture and *N*-glycans on the RBS [92]. Binding of free HKU1 S1 or free HKU1 S1^A^ not only to rat erythrocytes but also to mouse erythrocytes and to BSM cannot be detected by the standard method unlike other 9-*O*-Ac-Sia-binding β1CoVs, including HCoV-OC43 for which their free S1 and S1^A^ detectably bind to those erythrocytes and BSM [92,191]. The difference of HKU1 from other 9-*O*-Ac-Sia-binding β1CoVs was suggested to be due to receptor fine-specificity determined by elements 1 and 2. The effects of the internal part of the glycan structure, such as Siaα2,3/2,6Gal and LN repeats, on binding of HKU1 in comparison with other 9-*O*-Ac-Sia-binding β1CoVs should be further determined.

The cryo-electron microscopy structure of an HCoV-OC43 S trimer in complex with a 9-*O*-Ac-Me-Sia revealed that a sialoside-binding site was located at the surface-exposed groove of each S1^A^ monomer (Figure 5a) [193]. The sialoside-binding groove (Figure 5b,c) is formed by two loops, L1 consisting of 27-NDKDTG-32 and L2 consisting of 80-LKGSVLL-86 at the RBS edges, two hydrophobic pockets separated by the indole side chain of W90, P1 consisting of L85, L86 and W90 and P2 consisting of L80, W90 and F95 [193], and a residue, S87, interacting with L1 [92]. Substitutions of N27 having an H-bond with OA9 of the 9-*O*-acetyl carbonyl group, K81 forming H-bonds with O1 and N5 of Sia, or S83 containing an H-bond with O3 of Sia C1, with alanine and mutations of L80, L86 or W90 in hydrophobic pockets accommodating the 5-*N*-acyl moiety and the 9-*O*-acetyl-methyl moiety provided a mutant HCoV-OC43 S1^A^ that had lost the ability to bind to 9-*O*-Ac-6SLN. Substitutions at N27, T31, L80, K81, S83, L86 and W90 completely blocked the entry of pseudotyped VSVΔG particles harboring HCoV-OC43 S proteins into HEK293T cells. These results confirmed that residues in the surface-exposed groove are critical for interaction with the 9-*O*-Ac-Sia receptor and that their interactions are essential for mediating viral entry [193]. Interestingly, HCoV-OC43 S1^A^ recognized 9-*O*-Ac-Sia bound to Gal via α2,6 linkage. More research on binding specificity of both animal and human 9-*O*-Ac-Sia-binding β1CoVs to the internal part of the receptor in combination with analysis of 9-*O*-Ac-Sia-containing glycan structures expressed on host tissues and analysis of changes in the viral S1^A^ proteins could reveal which part of 9-*O*-Ac-Sia-containing glycans determines host/tissue tropism of β1CoVs and changes in the viral S1^A^ proteins associated with host/tissue tropism.

## 4. Protein Receptor-Dependent Recognition of Influenza A (H17–H18) Viruses and αCoVs, Lineage B βCoVs (β2CoVs)

### 4.1. Frugivorous Bat-Identified Influenza A (H17–H18) Viruses Use MHC Class II as a Receptor

The genomes of new IAV subtypes, H17N10, which was identified in liver, intestine, lung and kidney tissues and in rectal swabs, but not in oral swabs, of frugivorous yellow-shouldered bats (*Sturnira lilium*, family *Phyllostomidae*) in Guatemala [119], and H18N11, which was identified in intestine tissue and in rectal swabs of frugivorous flat-faced bats (*Artibeus planirostris*, family *Phyllostomidae*) in Peru [120] during the period from 2009 to 2010, were reported in 2012 and in 2013, respectively (Table 1). Based on aa sequences of H1–H18 HAs, both bat H17 and H18 HAs are more similar to H1–H2, H5–H6, H8–H9, H11–H13 and H16 in group 1 than to H3–H4, H7, H10 and H14–H15 in group 2 [198]. Analysis of the crystal structures of bat H17 and H18 HAs revealed that their overall structures retain possession of typical IAV HA molecules including the receptor binding site (RBS). However, bat HAs showed no binding to any of 610 diverse structures of glycans including more than 100 sialylated glycans with either α2,3, α2,6, α2,8 or mixed linkages. Detailed structural analysis (Figure 3d) revealed that at least four residues unique to bat H17 and H18 HAs seem to substantially reject a sialylated glycan from the bat RBS. (i) Highly conserved Y98 anchoring Sia is replaced by F98 in bat HAs. (ii) Highly conserved Q226 in H1 HAs and Q/L/I226 (a determinant of sialyl linkage specificity) in H3 HAs is replaced by H226 in bat HAs. (iii) The large residue D228 in bat HAs would make a steric clash with the side chain of Sia. (iv) The other large negatively charged residue D136 in bat HAs, possibly the most important residue, would provide electrostatic repulsion to a negatively charged sialylated glycan. Both structural and functional studies on bat HAs strongly confirmed that bat H17 and H18 HAs do not bind to sialylated glycans [120].

In 2019, human leukocyte antigen DR (HLA-DR) isotype, one of the three major MHC class II isotypes, found on susceptible cell lines including MDCKII clone no. 1, human U-87MG cells, human Calu-3 cells and human haematopoietic cancer cells was shown by the following findings to be required for mediating entry of pseudotyped viruses harboring H17 HAs [63] or H18 HAs [62] into a mammalian cell: (i) knockdown of HLA-DRA or HLA-DR α-chain or cell preincubation with an HLA-DRA-targeting antibody significantly reduced both H17- and H18-pseudotyped virus infections, (ii) ectopic expression of HLA-DR in non-susceptible cell lines rendered them susceptible to both H17- and H18-pseudotyped virus infections, (iii) expression of HLA-DR from other mammals, including different bat species, pigs and mice, or from chickens makes cells susceptible to H18-pseudotyped virus infection and (iv) intranasal infection of mice with H18N11 virus led to viral replication in the upper respiratory tracts of the mice. While these findings suggested possible spread of bat H17 and H18 viruses to other vertebrates, recent studies have shown that wild-type H18N11 infection is restricted to bats and that H18N11 has poor replication in mice and ferrets [199]. The potential of these viruses to spread to and infect other animals and the tissue tropism within other animals remain unclear. Characterization of direct binding of H17 and H18 HAs to MHC-II is also required. 

### 4.2. α HCoV-229E Uses hAPN Receptors

HCoV-229E was first recorded in the mid-1960s by two different groups in different countries: (i) Tyrrell and Bynoe [200] isolated the virus in a research unit in Salisbury, Wilts, England, and most of their work was done with a nasal swab of number B814 from a volunteer boy with a common cold and (ii) Hamre and Procknow isolated the virus from five medical students, including four students with mild upper respiratory illnesses (URIs) and one healthy student, using acute URI specimen number 229E as the prototype strain in the University of Chicago, United States [201]. Recently, investigation of the ancestral origins of the virus revealed that HCoV-229E has a high genetic identity to GhanaBt-CoVGrp1, a member of the bat CoV group (lineage) 1 that was isolated from fecal samples but was not found in oral swabs from insect-eating leaf-nosed bats, *Hipposidero (H.) caffer (cf.) ruber* [125,126] and *H. abae*, but not *H. jonesi* and *H. cf. gigas* [126], in Ghana, Africa. Results of molecular clock analyses indicated that HCoV-229E and GhanaBt-CoVGrp1 shared an old ancestor in approximately 1686–1800 C.E. [125]. In generic CoV RT-PCR screening, nasal swabs, but not fecal samples, from dromedary camels in Kenya and the Kingdom of Saudi Arabia (KSA) were positive for HCoV229E–related CoV (camelid-229E CoV), suggesting that the viruses are endemic in dromedaries. The results of genomic and phylogenetic analyses suggested that both human-derived and dromedary-derived CoVs are monophyletic [202]. The findings provide important implications for the emergence of HCoV-229E evolving from HCoV-229E-related CoVs (bat-229E CoVs) in bats of the genus *Hipposideros* in Africa as natural hosts [125,126] via dromedary camels in Africa and KSA as intermediate hosts [202].

**Figure 6:**
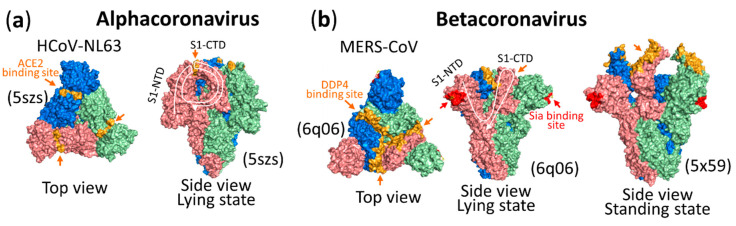
Structural comparison of αCoV and βCoV spike (S) proteins. Top and side views of homotrimeric S proteins, which are colored in salmon, marine and pale green, of αCoV (HCoV-NL63, pdb: 5szs [204]) (**a**) and βCoV (MERS-CoV, pdb: 6q06 [148] and 5x59 [205]) (**b**). Notably, a difference in folding of the S1 subunit (white arrow) of each monomer of αCoV and βCoV S proteins results in a difference in positions of the S1-CTD subdomain, next to the S1-NTD subdomain in αCoV but substantially separated from S1-NTD in βCoV. Consequently, the αCoV S trimer has a simple intra-subdomain packing, whereas βCoV has an intricate cross-subdomain packing as shown in the top and side views. S1-NTD is located on the external surface of the S1 trimer. S1-NTD, which functions as a receptor binding site, typically recognizes a host sugar receptor, except for MHV S1-NTD, which recognizes CEACAM1. In contrast, S1-CTD is located on the internal surface of the S1 trimer in the lying state. S1-CTD can undergo dynamic conformational changes to the standing state for efficient binding to its receptor. After binding, S1-CTD will be stabilized in the standing conformation. So far, S1-CTD, which functions as a receptor binding site, has been found to bind to a host protein receptor.

As in the case of α HCoV-NL63 virus shown in Figure 6a, α 229E viruses use their S1-CTDs on the top center of each S1 monomer of the S trimer to bind to host receptors. It is notable that about 396 amino acids at the N-terminus of the S protein of ancestral bat-229E CoV, which causes gastrointestinal tract infection, are deleted in HCoV-229E and camelid-229E CoV, which cause respiratory tract infection. Although this N-terminal spike deletion is not involved in the receptor binding site (RBS), it was presumed that the deletion is associated with the change in CoV tissue tropism from the gastrointestinal tract to the respiratory tract [203]. Further studies on its location on the spike 3D structure and on the mechanism of its function, which is thought to support virus replication in the bat gastrointestinal tract, as well as how the deletion helps the virus to replicate in the camel and human respiratory tracts may lead to an understanding of the molecular mechanisms of infection in the gastrointestinal and respiratory tracts. An understanding of the molecular mechanisms might lead to future prevention of gastrointestinal and respiratory infections. 

**Figure 7:**
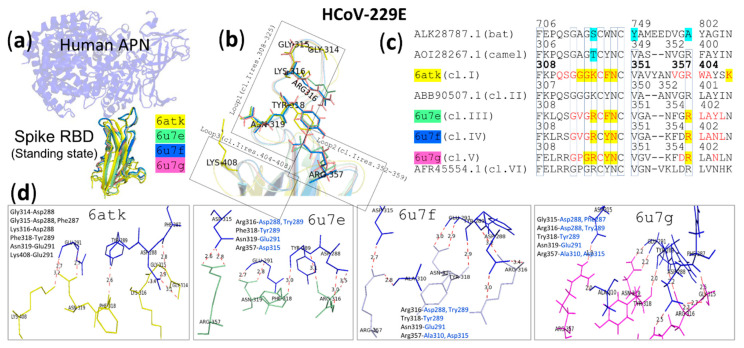
Comparison of receptor binding sites of six classes of S1-CTD RBDs of HCoV-229E variants. (**a**) Superimposition of pdb-available four classes (pdb: 6atk-class I (yellow), 6u7e-class III (pale green), 6u7f-class IV (marine) and 6u7e-class V (salmon)) of the S1-CTD RBDs of HCoV-229E variants in complex with human APN (transparent blue). (**b**) Positions of important aa residues in receptor binding sites (RBSs) of the four classes of the S1-CTD RBDs that have direct interactions with human APN. (**c**) Alignment of aa sequences of six classes of the S1-CTD RBDs. Residues in the S1-CTD RBDs facing the human APN receptor, called RBSs, are shown in red. The residues in RBSs directly interacting with residues in the APN receptor are highlighted in yellow. (**d**) Direct interactions between aa residues in human APN (blue sticks) and aa residues in RBSs of each S1-CTD RBD class. The bond lengths are shown on the dashed lines in angstrom units. 6atk is from [83], and 6u7e, 6u7f and 6u7g are from [194].

To enter cells, 229E viruses must bind to aminopeptidase N (APN) receptors (Figure 7a), dimeric glycoproteins that protrude from the epithelial cell surface (Figure 4b). Analysis of the crystal structures of HCoV-229E from four of six different classes that replaced each other in the human population [83], classified by an RBD sequence variant (aa 302–417), in complex with human APN (hAPN) revealed locations of a viral binding site (VBS) on hAPN (Figure 4b) and a receptor binding site (RBS) on the viral RBD (Figure 7b). The RBS is composed of residues with red letter codes (Figure 7c) in loops 1, 2 and 3 (residues 308–408 based on class I numbering, Figure 7b) on a six-stranded β-sheet peptide (Figure 7a). It should be noted that these loops are immunogenic [206]. The virus diverged into different classes with highly variable residues in the exposed loop sequences in order to evade neutralization by antibodies as observed by using IgG1 monoclonal antibodies against class I, a reference strain, in a viral infection inhibition assay [83]. Results of surface plasmon resonance binding assays showed that these virus variants had differences in binding affinity for hAPN receptors [83]. These differences in binding affinity might be for optimal escape and entry due to adaptation and selection of the virus to continue circulation under the condition of host environment pressure.

Mutagenesis of class I revealed that a C317S/C320S double mutation within loop 1 abolished binding to hAPN, suggesting that the C317-C320 disulfide bond is important for loop 1 folding and interaction with hAPN. While an F318A mutant showed a 13-fold reduction in affinity, both N319A and W404A mutations lead to a loss of hAPN binding [83]. Alignment of the RBS aa sequences of 6 classes of HCoV-229E with bat-229E CoV and camelid-229E CoV (Figure 7c) showed that 6 amino acids, F308, G313, G315, C317, C320 and N319 (based on class I numbering), are highly conserved among different classes and hosts. V351 and R359 are conserved in respiratory-transmitted viruses, HCoV-229E and camelid-229E CoV, but not in an oral-fecal-transmitted virus, bat-229E CoV. Amino acids at positions of K/R316, F/Y318 and N319 were observed in all 4 crystal structures to form hydrogen bonds with the receptor, principally stabilizing viral-receptor interactions (Figure 7d). However, the observation that bat- and camelid-229E CoVs contain S/T with a polar uncharged side chain instead of K/R316 with a longer charged side chain suggests that the amino acid at this position may be responsible for the change in receptor binding specificity from bat/camel to human APN receptors that is required for interspecies transmission. 

Viral binding sites on hAPN, based on interface residues of hAPN (red letter codes in Figure 4b) to 4 classes of HCoV-229E RBD [194] were aligned with bat *H. armiger* APN, due to the unavailability of APN of *H. cf. ruber* and *H. abae*, which are known to be bat hosts of HCoV-229E-related CoVs, and *Camelus dromedarius* APN. E291N, K292E, Q293T triple mutations generating a glycosylation site at position 291 in the hAPN abolished binding to all 6 HCoV-229E RBD classes [83], being in agreement with the finding that E291 plays a critical role in the formation of hydrogen bonds with N319 of the HCoV-229E RBD. Both E291 of hAPN and N319 of HCoV-229E RBDs are highly conserved among different hosts. In addition to E291, several amino acids on the viral binding site including A208, L243, T244, S285 E286, F287, V290, W303, P360, A308, G312, G314, L318 and N319 (based on hAPN numbering) are highly conserved among different hosts, and these amino acids on APN may therefore play a role in interspecies transmission of HCoV-229E-related CoVs, a possibility that should be further investigated. The specific binding of HCoV-229E-related CoVs to their host APN should be governed by other amino acids that are not conserved among different host species. These amino acids might be (i) K241, D242, and A310 in human and camel APNs, which are different from those in bat APN that carries S241, N242 and K310, (ii) D288, I309, A311, and D315, which are different from those in bat and camel APNs that carry T288, T309, E311, and I315, and (iii) Y289 and K292, which are different from those in bat APN carrying A289 and E292 and camel APN carrying C289 and G292. Mutations in hAPN, D288A, Y289A, V290G, I309A and L318A, resulted in hAPN binding affinity to HCoV-229E RBD, and the V290G mutant showed the greatest reduction (30-fold reduction) of binding affinity, indicating receptor binding specificity of the HCoV-229E RBD [83]. An understanding of the alteration in 229E receptor binding specificity should lead to prevention of virus infection and transmission. 

### 4.3. α HCoV-NL63, β2 SARS-CoV and β2 SARS-CoV-2 Use hACE2 Receptors

#### 4.3.1. α HCoV-NL63

HCoV-NL63 was first isolated from the culture supernatant of tertiary monkey kidney cells inoculated with a nasopharyngeal aspirate specimen, no. NL63, obtained from a 7-month-old baby girl with bronchiolitis and conjunctivitis in Slotervaart Hospital, Amsterdam, The Netherlands in 2003 and it was reported in 2004 [207]. In 2005, a novel strain, HCoV-New Haven (HCoV-NH), was identified by RT-PCR from RNA respiratory specimens collected from children less than 5 years of age from 2002 to 2003 in Yale-New Haven Hospital, New Haven, Connecticut, US. HCoV sequence comparisons revealed that HCoV-NH is likely to be the same species as HCoV-NL63, suggesting worldwide distribution of respiratory tract disease caused by HCoV-NL63, particularly in children [208].

In 2010, metagenomic analysis of viruses from feces, oral swabs, urine and tissues of 3 North American bat species, including big brown bats (*Eptesicus fuscus*), tricolored bats (*Perimyotis subflavus*) and little brown bats (*Myotis lucifugus*) in the Ridge and Valley physiographic province, by Appalachian Laboratory of the University of Maryland Center revealed that HCoV-NL63-related CoV, named Appalachian Ridge CoV strain 2 (ARCoV.2), existed in feces of tricolored bats in the family *Vespertilionidae* [209]. The results of molecular clock analysis suggested that HCoV-NL63 shares a common ancestor with ARCoV.2 with a most recent common ancestor (MRCA) of approximately 1190–1449 C.E [210]. In 2017, BtKYNL63-9a was reported to be the most closely related to HCoV-NL63 among three HCoV-NL63-related CoVs that were identified in fecal specimens collected between 2007 and 2010 from African trident bats (*Triaenops afer*) in the family *Hipposideridae* in Kenya and to be more closely related to HCoV-NL63 than to ARCoV.2 [127]. However, the genetic distance between HCoV-NL63 and ARCoV.2 or between HCoV-NL63 and BtKYNL63-9a is too large, and both ARCoV.2 and BtKYNL63-9a are therefore classified as not conspecific with HCoV-NL63 [211]. Based on the results of phylogenetic analyses, all proteins of HCoV-NL63 were found to cluster with the *Triaenops* bat NL63-related group, except for the S protein, which was nested within the *Hipposideros* bat 229E-related group, suggesting a chimeric genome of HCoV-NL63. Genome comparison indicated that there are two recombination breakpoints in the S gene: (i) near the 5′ end and (ii) at around 200 nucleotides upstream of the 3′ end. These data suggested that the conspecific ancestor of HCoV-NL63 is a recombination virus that emerged through co-infection between the *Triaenops* bat NL63-related CoV and the *Hipposideros* bat 229E-related CoV [127]. Thus, the recombinant virus should exist in bats or in an intermediate host (currently unknown for HCoV-NL63, probably a terrestrial mammal) that should be further identified. In addition, investigation of the relationship between *Perimyotis* ARCoV.2 and *Triaenops* bat NL63-related CoV may contribute to an understanding of the evolutionary origin prior to emergence of the recombinant ancestor of HCoV-NL63. 

#### 4.3.2. β2 SARS-CoV

SARS-CoV caused an epidemic outbreak of severe acute respiratory syndrome (SARS), which was first reported in November 2002 in Foshan, Guangdong in South China and it spread quickly from late February 2003. A global alert was issued by the WHO in March 2003, and it was declared to be contained in July 2003 [212,213]. The cumulative number of confirmed cases of SARS in the global epidemic from 1 November 2002 to 11 July 2003 was 8437 with 813 deaths (case fatality ratio of 9.6%) in 26 countries (29 areas) of 6 WHO regions [6]. In the post-global epidemic of SARS, 15 additional cases with one death from reappearance four times were reported during the period from December 2003 to May 2004. One reappearance with four cases in which only mild symptoms occurred was related to a restaurant serving palm civet meat (three cases) and house rats (one case) in Guangdong, China [214], indicating reintroduction of animal viruses into humans and the importance of effective surveillance for zoonotic diseases [215]. The other three reappearances were related to laboratory accidents in Singapore, Taiwan and China. The viruses from Singapore and Taiwan laboratories were not transmitted to others, while two infected cases from a Beijing laboratory subsequently spread to seven others with close contact, resulting in one death. This evidence indicated the importance of biosafety and biosecurity in a laboratory. Since the announcement by the WHO on 18 May 2004 that the SARS outbreak in China was contained, no SARS case has been reported [216]. 

Since reemergence of SARS from an animal virus could happen at any time, an understanding of the molecular evolution of the virus causing the past global epidemic may help to control future SARS outbreaks. Most of the human cases at the beginning of the SARS epidemic were caused by exposure to market animals (zoonotic source) [217]. Although more than 10 mammalian species, but no avian species, were discovered to be susceptible to infection with either SARS-CoV or SARS-related CoVs (SARSr-CoVs), both the number of animals traded in Guangdong, China, and the detection rate were higher in Himalayan or masked palm civets (*Paguma larvata*) than in other animals. Moreover, there were close matches between sequences of civet viruses and sequences of human viruses from each human outbreak, including the 2002–2003 epidemic and the 2003–2004 episode. These findings suggested that palm civets are important intermediate hosts for transmission of the virus to humans [218], probably through direct/indirect contact or inhalation of contaminated materials/droplets. Screening of SARSr-CoVs in palm civets by real-time RT-PCR and nested RT-PCR for detection of the N gene and P gene revealed the presence of the virus in rectal and/or throat swabs [100]. Major genetic variations in the S gene were found by genomic sequence analyses of civet viruses (SARSr-CoVs) and human viruses (SARS-CoVs), indicating that changes in the S gene are likely to be critical for shifting the virus from civet-to-human to human-to-human transmission that caused the 2002–2003 epidemic [214]. Kan et al. [100] analyzed all available SARS S gene sequences and found 27 signature nucleotide variations (SNVs). Based on SNVs and a phylogenetic tree of the SARS S genes from animal and human viruses, the viruses were divided into four groups. (i) Viruses without SNVs in the S gene, called a prototype group, were isolated from raccoon dogs and a palm civet but not from humans, suggesting that they can cause only animal-to-animal transmission. (ii) Viruses with two to seven SNVs generate up to six aa changes at the positions of 147, 228, 240, 479, 821 and 1080. These viruses were isolated from palm civets and from mild symptomatic patients (so-called low-pathogenic group) during the 2003–2004 episode, indicating that the virus in a palm civet can acquire the ability to infect humans. (iii) Viruses with 17 to 22 SNVs cause further eleven aa changes at the positions of 360, 462, 472, 480, 487, 609, 613, 665, 743, 765, and 1163. These viruses were isolated from palm civets and raccoon dogs in 2003 and from patients with severe symptoms (so-called high-pathogenic group) who had close contact with infected animals or patients in the early-phase epidemic (16 November 2002 to 30 January 2003). This evidence indicated the possibility that animal species other than civets may be intermediate hosts transferring the animal virus to humans. The SNVs of viruses in this group indicated that the virus in palm civets and raccoon dogs can evolve not only to infect humans but also to spread from one human to other humans by close contact, indicating the possibility that these animals and humans may share similar receptor structures. (iv) Viruses with 25 to 27 SNVs cause up to four aa further changes at the positions of 227, 244, 344, and 778. These viruses in this group were isolated from patients with severe symptoms in the middle phase (beginning on 31 January 2003: hospital phase) and late phase (beginning on 21 February 2003: hotel phase) of the 2003 epidemic that was responsible for the global outbreak, so-called large epidemic outbreak group [100]. 

While SARSr-CoVs are not widespread in farmed palm civets [100] and wild palm civets [219], SARSr-CoVs collected from bat anal/fecal swabs show high genetic diversity and are widespread in wild Chinese horseshoe bats in the genus *Rhinolophus* (*Rhinolophus sinicus* [95], *R. pussilus* (seropositive in blood specimens), *R. pearsoni*, *R. macrotis* and *R. ferrumequinum* [220]). Based on these observations, bats were believed to be the natural reservoir of SARS-CoVs. However, phylogenetic relationships suggested that none of the virus isolates from wild bats are direct ancestral viruses of SARS-CoV [130]. It is most likely that SARS-CoV evolved through recombination of bat SARSr-CoVs [128,129,130]. 

#### 4.3.3. β2 SARS-CoV-2

SARS-CoV-2, which shares about 80% nt sequence identity with its elder cousin SARS-CoV [221], causes an acute respiratory disease that was officially named coronavirus disease 2019 (COVID-19). It is a novel coronavirus (2019-nCoV) that was first recognized in December 2019 in Wuhan, Hubei in Central China [222]. Different from SARS-CoV, with a mortality rate of 9.6% in cases of infection, SARS-CoV-2 generally causes mild to moderate disease, but it can also lead to severe disease and death in some cases [223,224]. Keeping the infected hosts alive enabled SARS-CoV-2 to adapt to humans with efficient human-to-human transmission. It spread rapidly worldwide, finally causing the COVID-19 pandemic, with outbreaks sustained in more than one WHO region [154], on 11 March 2020. As of 8 September 2020, the pandemic is ongoing and has caused 27,205,275 confirmed cases reported from 182 countries and 33 territories and reported from international conveyances throughout six WHO regions, resulting in 890,392 deaths, giving a tentative 3.3% case fatality rate [225]. 

Analysis of whole-genome sequences showed that SARS-CoV-2 shares about 96.1% identity to a bat SARSr-CoV isolated from *Rhinolophus affinis* (Ra) in Yunnan, China in 2013 (BatCoV RaTG13) [132,133], 93.3% identity to a bat SARSr-CoV isolated from *Rhinolophus malayanus* (Rm) in Yunnan (YN), China in 2019 (BetaCoV/Rm/Yunnan/YN02/2019, RmYN02) [133], and 87.8% and 87.6% identity to two bat SARSr-CoVs, BatCoV RpZC45 and BatCoV RpZXC21, respectively, detected in *Rhinolophus pusillus* (Rp) bats from Zhoushan City (ZXC or ZC), Zhejiang Province, China in 2015 [226]. As was the case for SARS-CoV, SARS-CoV-2 is likely to have originated from Chinese horseshoe bats in the genus *Rhinolophus*. Although there is high sequence identity between the longest encoding gene region (1ab) of RmYN02 and 1ab of SARS-CoV-2 (nt 97.2%, aa 98.8%), there is low sequence identity between the SARS-CoV-2 S RBD and RaTG13 S RBD (nt 85.3%, aa 89.3%) or RmYN02 S RBD (nt 61.3%, aa 62.4%) [133]. The RBD is a major determinant of host range and it is therefore likely that there is an intermediate host facilitating a bat SARSr-CoV to acquire efficient ability to infect humans. 

Although the first outbreak occurred in Wuhan, animal specimens from the Huanan seafood wholesale market in Wuhan, which sells both live and dead animals including bats, civets, snakes, poultry, pigs and dogs, were negative for SARSr-CoVs [226]. Snakes and canids (dogs) were presumed to be intermediate hosts of SARS-CoV-2 based on sequence analysis of the relative synonymous codon usage (RSCU) bias between SARS-CoV-2 and animal host species [227] and based on analysis of zinc finger antiviral protein (ZAP) expression in animal host species and tissues that drive CoV evolution to have a low-CpG (5′-Cytosin-phosphate-Guanine-3′) viral genome [228], respectively. However, SARSr-CoV-2 has not been isolated from snakes and it is unlikely that the viruses can cross the species barrier from bats, warm-blooded mammals, to humans via snakes, cold-blooded reptiles. Although results of RT-PCR and serological tests confirmed SARS-CoV-2 infections in dogs, the infected dogs were with infected owners and thus humans are likely to have transferred the virus to their pets [101]. Cats, tigers and lions that were cared for by infected owners/zookeepers were also reported to have tested seropositive for SARS-CoV-2, suggesting human-to-animal transmission of COVID-19 [101]. Experimental studies showed that pigs, chickens and ducks were not susceptible to SARS-CoV-2, that dogs had little susceptibility and that both ferrets and cats were highly susceptible [229,230]. Experimental studies showed that there is little transmission of SARS-CoV-2 among ferrets but that cats have the potential for airborne transmission of the virus between them [230]. However, no cat-SARSr-CoV-2 has been isolated. Furthermore, there has so far been no evidence of transmission of SARS-CoV-2 or SARSr-CoV-2 from these SARS-CoV-2-susceptible animals, including canines, ferrets and felines, to humans [101,229]. Nonetheless, surveillance of both infection and dissemination of SARS-CoV-2 should be implemented. SARSr-CoV-2 has been isolated from smuggled Malayan pangolins (*Manis javanica*), but it has not been isolated from Chinese pangolins (*Manis pentadactyla*) seized in Guangxi (GX) and Guangdong (GD) in southern China [103,226,231]. Whole-genome comparison indicated that pangolin-SARSr-CoVs have a significant sequence difference from SARS-CoV-2 sequences, suggesting that current pangolin-SARSr-CoV isolates are unlikely to be the virus directly transmitted to cause SARS-CoV-2 outbreaks in humans. However, all of the studies [103,133,226,231] showed high aa sequence identities (97.4%) between pangolin SARSr-CoV (pangolin/GD/2019 with a single consensus sequence merged from the GD/P1L and GD/P2S sequences) S RBD and SARS-CoV-2 S RBD. Thus, SARSr-CoV from Malayan pangolins may be able to infect humans or may provide an RBD gene region to a coinfected CoV. Malayan pangolins may serve as a vessel to generate a CoV with human receptor-binding potential due to the high aa sequence similarity between pangolin and human ACE2 receptors (84.8%) [231], suggesting the need for pangolin surveillance for public health. The continued search for a SARS-CoV-2 intermediate host is essential for understanding the emergence of the COVID-19 pandemic and for future prevention and control of zoonotic CoV-related diseases.

#### 4.3.4. Receptor Binding Specificity of α HCoV-NL63, β2 SARS-CoV and β2 SARS-CoV-2

Results obtained by direct biochemical methods and X-ray crystallographic studies showed that α HCoV-NL63 [84,232], β2 SARS-CoV [233] and β2 SARS-CoV-2 [195] use their S1-CTD RBD to bind to a host receptor, angiotensin-converting enzyme 2 (ACE2, a zinc peptidase), that is essential for virus entry into cells. As shown in Figure 4b, ACE2 is a homodimeric type I transmembrane protein having an orientation with the N-terminus outside and the C-terminus inside the cytoplasm [195]. The virus-binding site (VBS) of these three CoVs is not the peptidase active site but the outer surface of the ACE2 N-terminal lobe. Analyses of cocrystal structures between RBDs of HCoV-NL63 (pdb: 3kbh [232]), SARS-CoV (pdb: 2ajf [234]) or SARS-CoV-2 (pdb: 6m0j [235]) and the human ACE2 (hACE2) receptor indicated aa residues covering the CoV–ACE2 interfaces, divided into a common region of hACE2 recognized by all three ACE2-recognizing CoVs (a hotspot region) and unique regions bound by HCoV-NL63, SARS-CoV or SARS-CoV-2 (Figure 4b, middle row). Evidence indicating that HCoV-NL63 and SARS-CoV bind to the same hotspot region on hACE2 and that their binding is important for infection was obtained from infection inhibition studies showing that the SARS-CoV RBD can inhibit lentivirus infections mediated by the S protein of either SARS-CoV or HCoV-NL63 into hACE2-expressing HEK293T cells [236]. Likewise, the use of the HCoV-NL63 RBD as a competitive inhibitor can inhibit infections of murine leukemia viruses (MLVs) mediated by SARS-CoV S protein into hACE2-expressing HEK293T cells [237]. In addition, aa changes in the hotspot region in hACE2, either L353A or D38A substitution, resulted in a significant reduction of binding interactions between the SARS-CoV or HCoV-NL63 RBD and hACE2 and reduction of MLV infections mediated by SARS-CoV or HCoV-NL63 S protein [237]. The results of these studies suggested that the hotspot region on the hACE2 VBS is a potential target for development of drugs against ACE2-binding CoVs. It should be noted that MLN-4760, an ACE2 inhibitor that binds to the ACE2 catalytic center and induces hACE2 conformational changes, did not affect interactions of SARS-CoV S1 with the hACE2 surface and did not affect SARS-CoV S protein-mediated infection. Likewise, binding of SARS-CoV S1 to hACE2 did not affect hACE2 catalytic activity [238].

**Figure 8:**
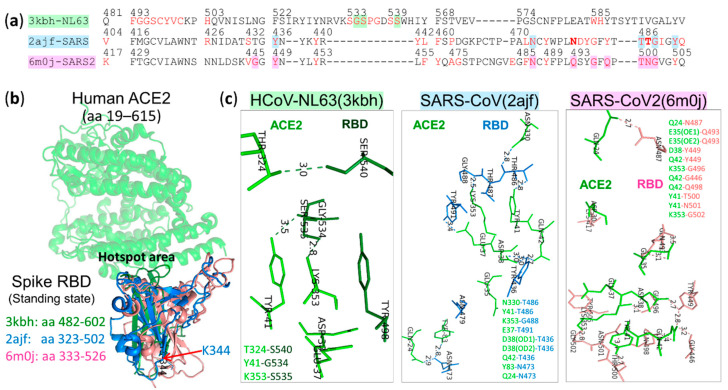
Comparison of receptor binding sites in S1-CTDs of HCoV-NL63, SARS-CoV and SARS-CoV2 binding to human ACE2. (**a**) S1-CTD receptor binding domains (RBDs) of HCoV-NL63 (pdb: 3kbh [232], forest), SARS-CoV (pdb: ajf [234], marine) and SARS-CoV-2 (pdb: 6m0j [235], salmon) in complex with human ACE2 (transparent green). (**b**) Direct interactions between amino acids of human ACE2 and those of the S1-CTD RBD of the indicated CoV. The bond lengths in angstroms are indicated on the dashed lines. (**c**) Alignment of aa sequences of the S1-CTD RBDs of the three CoVs binding to human ACE2. Amino acid residues in the S1-CTD RBDs facing the ACE2 receptor are colored in red. Residues in the S1-CTD RBDs of HCoV-NL63, SARS-CoV and SARS-CoV-2 directly interacting with residues in the ACE2 receptor are highlighted in green, blue and purple, respectively.

The aa sequences of hACE2 were aligned with aa sequences of ACE2 orthologues of possible natural reservoirs and possible intermediate hosts. Only the aa sequences corresponding to the hACE2 interface are shown in Figure 4b. Adaptation of each zoonotic virus to interact with aa residues at the hACE2 binding interface is critical for efficient transmission among humans. The hACE2 residues K31, D38, Y41 and K353 are important host determinants of adaptation of civet SARSr-CoV to human SARS-CoV (Figure 4b), and viral S1 RBD residues at the positions of 479 and 487 are important determinants of SARS-CoV binding preference (Figure 8a–c). The K479N mutation from civet to human viral S1 RBD can accommodate K31 on hACE2 [238]. The S487T mutation from civet to human viral S1 RBD can accommodate a hydrophobic pocket between Y41 and K353, neutralized by D38, on the hACE2 receptor for efficient interactions [234]. These findings agree with results obtained by Kan et al. [100] suggesting that viruses with SNVs leading to aa changes at these two positions are able to be transmitted from animals to infect humans and from humans to humans by close contact. However, the roles of the additional 4 aa substitutions at position 344 in the RBD but outside the RBS (Figure 8b) and positions 227, 244 and 778 outside the RBD in viruses isolated from patients during the global epidemic [100] in human-to-human transmission remain unknown. 

Crystal structure analysis indicated that the SARS-CoV-2 RBD to which hACE2 binds is almost identical to the SARS-CoV RBD [235]. Later, hACE2 amino acids at or near the RBD/ACE2 interface (Figure 4b) that could affect RBD/ACE2 binding were used for screening the capability of ACE2 of various animals used by SARS-CoV and SARS-CoV-2. ACE2 of possible SARS-CoV and SARS-CoV-2 intermediate hosts, masked palm civet and Malayan pangolin, respectively, and ACE2 of many mammals including cats, dogs, cows, buffalos, goats and sheep, but not rats (*Rattus norvegicus*), were predicted to be potentially recognized by SARS-CoV and SARS-CoV-2 [239,240], supporting the finding that rat ACE2 has less efficiency for binding to the SARS-CoV S1 domain and is less susceptible to SARS-CoV S protein-mediated infection [241]. However, young female Fischer 344 (F344) rats of 4 weeks of age were shown to be susceptible to infection with SARS-CoV by intranasal inoculation [242]. Western blot analysis showed that ACE2 expression in Sprague Dawley rats decreased with aging without a gender difference [243]. However, it remains unknown whether there is a difference in ACE2 sequence depending on the age of rats and whether there are differences in ACE2 expression and sequence depending on the rat strain. Based on ACE2 residues 31, 35, 38, 82 and 353, Chinese horseshoe bats, which are thought to be a natural reservoir, can be divided into two groups [240]. First, bat ACE2 of SARSr-CoV–RT-PCR-positive *R. ferrumequinum* (bat Rf) [220] was predicted not to have the ability to bind to either SARS-CoV-2 or SARS-CoV S protein. Second, bat ACE2 of SARSr-CoV–seropositive and –RT-PCR-positive *R. pearsonii*, *R. macrotis* [220], and SARSr-CoV–RT-PCR-positive *R. sinicus* [95] was predicted to be able to bind to both SARS-CoV-2 and SARS-CoV S proteins. Based on residues 20, 31, 41, 68, 83, 353, 355, 357 and 383, *R. sinicus* ACE2 was confirmed to have the potential to be used by SARS-CoV-2 [239]. These findings indicate the possibility of cross-species transmission of the virus from humans to animals carrying similar host receptor sequences, although other host factors, such as target organ temperature and cellular proteins interacting with the virus, may be involved in host range restriction of the virus. Thus, surveillance of transmission both back and forth between humans and animals is needed. The susceptible host range of HCoV-NL63, which causes mild respiratory disease [244], has not been determined. However, the host range of HCoV-NL63 might be similar to that of SARS-CoV and SARS-CoV-2, and thus a mixed infection of these different viruses to the same host cells may occur.

Although α HCoV-NL63, β2 SARS-CoV and β2 SARS-CoV-2 recognize the same ACE2 receptor and all bind to the hotspot region on ACE2 (Figure 4b, middle), they have aa differences in the RBS at the viral RBD interface (Figure 8a), suggesting that they have undergone convergent evolution for efficient ACE2 binding [232,235]. While SARS-CoV and SARS-CoV-2 have similar RBD structures with a concave surface, α HCoV-NL63 has no structural RBD homology to βCoV RBDs (Figure 8b) [232,235]. Thus, we superimposed hACE2 receptors (green) in complex with HCoV-NL63 (pdb: 3kbh), SARS-CoV (pdb: 2ajf) and SARS-CoV-2 (pdb: 6m0j) as shown in Figure 8b. However, since all three viruses interact with the same hotspot region on the hACE2 receptor, RBS residues of these different viruses occupy similar positions in the hotspot area. For example, S535/T487/N501 of HCoV-NL63/SARS-CoV/SARS-CoV-2 are located near K353 and Y41 of hACE2, while Y498/Y491/Y505 of HCoV-NL63/SARS-CoV/SARS-CoV-2 are located near K353, E37 and D38 of hACE2 (Figure 8c) [235,237]. By using surface plasmon resonance with a Biacore 2000/3000 instrument, equilibrium dissociation constant (*Kd*, smaller value indicating greater binding affinity) values between HCoV-NL63 RBD and immobilized hACE2 and between SARS-CoV RBD and immobilized hACE2 were determined to be 34.9 and 20.8 nM, respectively [232,237]. It should be noted that NL63-CoV RBD-hACE2 interactions have lower *koff* and *kon* values than do SARS-CoV RBD-hACE2 interactions, suggesting that NL63-CoV RBD/hACE2 complex has less electrostatic and more hydrophobic interactions [237]; three hydrogen bonds were observed in HCoV-NL63 RBD-hACE2 complex, but nine hydrogen bonds were observed in SARS-CoV RBD-hACE2 complex (Figure 8c). By surface plasmon resonance with a Biacore T200 instrument, the *Kd* values of SARS-CoV RBD-immobilized hACE2 and SARS-CoV-2 RBD-immobilized hACE2 were determined to be 31 nM and 4.7 nM, respectively [235]. As mentioned earlier, S487T and K479N substitutions in the civet SARSr-CoV RBS are critical for civet-to-human transmission [238]. It appears that both T487 and N479 are substituted by N501 and Q493, respectively, in the SARS-CoV-2 RBS. N501 in the SARS-CoV RBD and T486, but not T487, in the SARS-CoV RBD (Figure 8c) form a hydrogen bond with hACE2 Y41. It is likely that subtle differences between the SARS-CoV RBD and SARS-CoV-2 RBD in interactions with hACE2 are responsible for the difference in *Kd* values of the SARS-CoV RBD and SARS-CoV-2 RBD for hACE2 [235]. For example, while N479 in the SARS-CoV RBD does not cause hydrogen bond formation, its substituted Q493 in the SARS-CoV-2 RBD makes two hydrogen bonds with E35 of hACE2. K417 in the SARS-CoV-2 RBD provides a unique interaction with huACE2 D30 and a positive charged patch on the SARS-CoV-2 RBD, which is not found on the SARS-CoV RBD (Figure 8c) [235]. A virus with a great binding affinity, which can trigger infection efficiently, could be a factor of the rapid spread of SARS-CoV-2. Other factors may be involved in driving the rapid spread of the virus. For example, the presence of a polybasic (RRAR) site at the S1/S2 cleavage site found in SARS-CoV-2, but not in other βCoVs in lineage B, which is cleaved by furin pre-activating the viral S proteins during virus exit, reduces dependence of the viral S proteins on target cell proteases for virus entry and thus facilitates the virus infection [245].

## 5. Sialyl Glycan and Protein Receptor-Dependent Recognition of Lineage C β (β3) MERS-CoV

A novel CoV was first isolated from the sputum of a Saudi Arabian patient who died from acute respiratory distress syndrome (ARDS) and subsequent multiorgan dysfunction syndrome (MODS) in 2012 [246], and the novel CoV was named Middle East respiratory syndrome coronavirus, MERS-CoV, in 2013 [247]. Most infected patients present with atypical pneumonia that has the potential to progress to ARDS [104,247]. Although confined to the Middle East, mainly in Saudi Arabia, human MERS-CoV infection spread to 27 other countries from people traveling. As of May 2020, 2562 cases with 881 deaths have been reported to WHO, the case-fatality rate being approximately 34% [8]. New MERS-CoV infection cases caused by direct or indirect contact with infected dromedary camels (*Camelus dromedaries*) or by close contact with infected humans have continued to be reported, and MERS is thus still a disease of a global concern. By comparing ∼30-kb genome sequences, MERS-CoVs isolated from patients were reported to share more than 99% nt identity to dromedary MERS-CoVs [248]. Based on results of nt sequence and phylogenetic analyses, MERS-CoVs are closely related to several lineage C βCoVs/RNA detected in feces of different bat species but have 100% nt identity to a CoV gene fragment (only 182 nucleotides) isolated from *Taphozous perforatus* bat feces in Bisha, Kingdom of Saudi Arabia. Based on complete genome sequences that have been so far been determined, the most closely related virus sharing about 85.6% nt identity to MERS-CoV is NeoCoV isolated from *Neoromicia capensis* bat feces in South Africa [106]. Dromedary camels are the only confirmed hosts of zoonotic MERS-CoV leading to human infection [111]. MERS-CoV or its RNA can be detected in dromedary nasal swabs and lung tissue samples, and an experimental study showed that MERS-CoV appears to cause mild upper respiratory tract disease in dromedary camels [109,110], suggesting that dromedary MERS-CoV is transmitted through droplets (either droplet particles or droplet nuclei) and contact routes. These findings suggested that bats are ancestral reservoir hosts and dromedary camels are intermediate reservoir hosts of MERS-CoVs.

**Figure 9:**
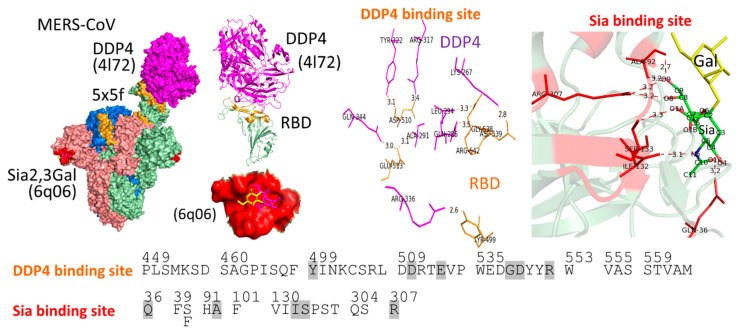
Binding of MERS-CoV S1-CTD to human DDP4 receptor (pdb: 4l72) and S1-NTD to Siaα2,3Gal receptor (pdb: 6q06). Middle: Cartoon diagram of MERS-CoV S1-CTD (pale green) in complex with human DDP4 (magenta) and surface diagram (red) of MERS-CoV S1-NTD in complex with Siaα2,3Gal receptor (Sia in magenta stick and Gal in yellow stick). Amino acids in the bright orange area of S1-CTD and in the red area of S1-NTD being a DDP4 binding site and a Sia binding site, respectively, are shown in the lower panel. Right: Direct interactions between amino acids of the DDP4 binding site and those of the DDP4 receptor and between amino acids of the Sia binding site and those of a Siaα2,3Gal receptor. The bond distance in angstroms of each direct interaction is shown on the dash lines. Amino acids involved in indirect interactions are highlighted in gray in the lower panel. 4l72, 6q06 and 5x5f are from [251], [148] and [205], respectively.

MERS-CoV infection is initiated by attachment of its homotrimeric S proteins to a host cell. By mass spectrometric analysis, dipeptidyl peptidase 4 (DPP4 or CD26) was identified to be a ∼110-kDa protein co-purified with MERS-CoV S1–Fc chimeric protein from the human liver (Huh-7) and African green monkey kidney (Vero) lysates. Pre-incubation of Huh-7 cells and primary human bronchial epithelial cells with anti-DPP4 immunoglobulin appeared to inhibit MERS-CoV infection. Based on these findings, MERS-CoV infection was suggested to occur via binding of MERS-CoV S1 to the cellular receptor DDP4 [249]. According to the site of virus infection in individual hosts, DDP4 was detected in the respiratory tracts of camelids and humans and was found to be rich in the intestinal tracts of pipistrelle bats, the hosts of *Pipistrellus* bat CoV HKU5 (Pi-BatCoV HKU5), which is closely related to MERS-CoV [108,131]. Like other protein-binding CoVs, except for MHV binding to the protein receptor CEACAM1 via S1-NTD [250] (Table 2), MERS-CoV binds to the outer surface of DDP4 via S1^B^ (S1-CTD) and it does not bind to the DDP4 catalytic pocket and does not require DDP4 catalytic activity (Figure 4b) [251]. In a cryo-EM study, each monomeric S1-CTD receptor binding surface was found to be buried in the tip of the CoV S trimer in the lying state (closed conformation, pdb: 6q06) and to be exposed in the standing state (open conformation, pdb: 5 × 59) (Figure 6b) readily bound by the receptor (Figure 9, left) [205]. Analysis of the crystal structure of MERS-CoV S1-CTD in complex with DDP4 (pdb: 4l72) revealed the location of the DDP4 binding site on MERS-CoV S1-CTD (Figure 9, left) and the viral S1-CTD binding site on DDP4 (Figure 4b) and provided information on interactions between the viral S1-CTD and DDP4 [251]. Eight H-bond formations were observed between residues on the viral S1-CTD and residues on the DPP4 as follows: (i) Y499 with R336, (ii) D510 with R317, (iii) D510 with Y322, (iv) E513 with Q344, (v) E513 with A291, (vi) G538 with Q286, (vii) D539 with K267, and (viii) R542 with L294 (Figure 9, middle).

Observation of agglutination of human erythrocytes by intact MERS-CoV particles led to the finding that Sia-binding activity of MERS-CoV is mediated by multivalent S1^A^ (S1-NTD) [112]. One hundred thirty-five glycan structures were used for determination of receptor binding specificity of multivalent MERS-S1^A^-containing nanoparticles by glycan arrays. It appeared that MERS-S1^A^ nanoparticles bind selectively to nonmodified Neu5Ac but not to Neu5Gc or (7,)9-*O*-acetylated Neu5Ac. No binding to Neu5Gc-terminated glycans was explained by steric hindrance of the extra hydroxyl group of Neu5Gc in the MERS- S1^A^ hydrophobic pocket formed by F39, F101, I131 and I132 [148]. For sialyl linkage specificity, MERS-CoV S1^A^ prefers α2,3 over α2,6 linkages, either short, sulfated, α2,3-linked mono-Sia Lewis^X^ or long, branched, α2,3-linked tri-Sia with four type 2 LN repeats. Pretreatment of Calu-3 human airway cells with neuraminidase inhibited infection of MERS-CoV, indicating that Sia is necessary for MERS-CoV infection. Accordingly, multivalent MERS-CoV S1^A^-containing nanoparticles were shown to specifically bind to infection sites in individual hosts: dromedary camel nasal epithelial cells, human type II pneumocytes in the alveolar wall that contains an abundance of α2,3-sialyl type 2 LN and a small amount of α2,3-sialyl-Lewis^X^ [19], and pipistrelle bat intestinal epithelial cells. Removal of Sia from cell surface glycans by treatment with neuraminidase abolished binding of the nanoparticles, confirming that MERS-CoV S1^A^ binds to infected cells via Sia recognition [108]. The cryo-EM structure of MERS-CoV S in complex with α2,3-SLN (pdb: 6q06) showed Sia binding in a surface-exposed groove (domain A) of each monomer (Figure 9, left). Analysis of the structure indicated the five residues Q36, A92, I132, S133 and R307 directly anchoring Neu5Ac as shown in Figure 9, right [148]. 

Recently, Qing et al. [113] demonstrated roles of S1^A^ domains binding to Sia receptors and S1^B^ domains binding to DDP4 receptors at different stages of virus infection. At the initial infection stage, MERS-CoV attaches to sialyl receptors on the host cell membrane and requires subsequent durable adherence to DDP4 receptors for infection. At later infection stages, MERS-CoV S proteins, which are not incorporated into progeny viruses, abundant on infected cell membranes attach to sialyl receptors on neighboring cells. This stage does not require DPP4 receptors but requires the cell surface protease TMPRSS2 for activating MERS-CoV S proteins for cell-cell fusions resulting in syncytial formation that rapidly spreads. These findings indicate the importance of Sia attachment playing roles in both initial and later infection stages and possibly determining the site of MERS-CoV infection since Sia attachment precedes DPP4 adherence at the initial infection stage.

## 6. Conclusions and Future Perspectives

Despite much effort in studies and preparedness from deadly viruses including HPAI, SARS and MERS outbreaks, the unexpected 2009 H1N1 strain and 2019 SARS-CoV-2 quickly spread worldwide and became pandemics in 2009 and 2020, respectively. Although H5 and H7 vaccines, either monovalent or bivalent forms, are used for protection of domestic birds [252], the ever-changing and highly reassorting HPAI H5 has continued to cause outbreaks in domestic birds and to be detected in dead wild birds [24]. HPAI H7N3 in poultry has been reported until now [24]. HPAI H5N6 human cases [253] and HPAI H7N9 human cases [254] were still reported in 2019. Several avian IAVs including H7N9, HPAI H5NX (X, NA subtypes) and H9N2 sporadically cross species to infect humans. MERS-CoV is still causing an endemic in dromedary camels and human infections [8] and SARS-like CoV is still circulating in animals, notably in palm civets and raccoon dogs [100]. Thus, it is very important to understand how a new pandemic arises. 

Historical data indicated that CoVs, which infect humans by using a specific host protein as a primary receptor, including HCoV-229E (APN), HCoV-NL63 (ACE2), SARS-CoV (ACE2), SARS-CoV-2 (ACE2) and MERS-CoV (DDP4), originated from bats, whereas HCoV-OC43 and HCoV-HKU1, which use Neu5,9Ac_2_, originated from rodents [255]. In contrast, IAVs infecting humans using sialyl glycans are thought to have originated from wild birds. The finding that some H16 HAs isolated from wild birds contain Y98F, being different from HAs of other Sia-binding IAVs but the same as bat non-Sia-binding HAs of H17 and H18 IAVs, and the finding that there are bat α2,3Sia-binding IAV isolates that are phylogenetically different from other identified IAVs with HA closest to mallard H9 viruses indicate the possibility of cross wild bird-to-bat and/or bat-to-wild bird transmission of IAVs [64]. Studies on evolutional changes of wild bird IAVs and bat IAVs may lead to clarification of the host origins of IAVs. An understanding of viral adaptation in the wild animals to recognize distinct receptors may lead to the establishment of strategies for efficient control of CoVs and IAVs. 

Data from genome analysis of the past three IAV pandemics, except for the first identified 1918 H1N1 pandemic that remains a mystery, demonstrated that a pandemic emerged from viral reassortment with a human virus gene segment(s) and a nonhuman HA gene segment producing a major viral antigen [19]. Extensive studies on receptor binding specificities of IAVs from different hosts [19,256] have indicated that a nonhuman HA gene must acquire mutations to recognize α2,6Neu5Ac for efficient human-to-human transmission, leading to the development of several techniques for monitoring and assessing IAV pandemic potential, including a viral NA-based assay [257], a glycan microarray assay [258], an evanescent-field-activated fluorescence scanner type glycan array [259] and a glycan strip test [260]. However, occasional direct pig-to-human [261] and human-to-pig [262] transmission of swine and human IAVs, respectively, both of which preferentially bind to α2,6 sialyl glycans, and some avian IAVs, including HPAI H5N1 [263], H7N9 [264], H9N2 [265], H7N2 and H7N3 [266], that were reported to have increased binding to human-type α2,6Neu5Ac have not yet caused a pandemic. In addition to monitoring the α2,3 to α2,6 binding shift of HA, a simple test for monitoring shifts in other factors, such as PB2, should be developed for monitoring the situation of a virus with pandemic potential. 

Different from IAVs having a segmented (-)ssRNA genome, CoVs have a nonsegmented (+)ssRNA genome. SARS-CoV and MERS-CoV originated through recombination in bats [267] and cross-species transmission to intermediate hosts, palm civets and dromedary camels, respectively, resulting in transmission to humans. Both palm civet SARSr-CoV and human SARS-CoV and dromedary camel MERS-CoV and human MERS-CoV have 99.8% nt sequence identity [217,268]. In contrast, a nonhuman virus that is almost identical to SARS-CoV-2 has not been identified so far, suggesting no widespread infections of a nearly identical SARS-CoV-2 in natural or intermediate hosts [269]. Based on current data, SARS-CoV-2 might have emerged from a quadruple recombination by which Yunnan bat RaTG13 (96.1% nt identity with SARS-CoV-2) might be its genome backbone [226], Yunnan bat RmYN02 (93.3% nt identity) probably gave the long replicase gene (1ab gene, sharing 97.2% nt identity) [133], pangolin SARS-like-CoV-2/Guangdong might have provided an RBD motif-coding gene (97.4% aa similarity) [231], and an unidentified bat virus might have donated a gene region coding a multibasic (furin, PRRAR motif) cleavage site [133]. Similarly, the 2009 H1N1 pandemic is a quadruple reassortant IAV that acquired gene segments from human IAV (PB1 gene), avian IAV (PB2 and PA genes), classical swine IAV (H1, NP and NS genes) and Eurasian avian-like swine IAV (N1 and M genes) [4].

The ability of a virus to adapt to a human environment, including human immunity and drugs, enables it to seasonally circulate in humans. Highly transmissible SARS-CoV-2, which has the same ACE2 receptor as that of HCoV-NL63, may continue to be a seasonal CoV and may undergo recombination with HCoV-NL63 during mixed infection in the same cell. While there is no vaccine for and no specific drugs for treatment of mild common cold disease caused by HCoVs, many efforts are being made to develop both vaccines and drugs for the pandemic SARS-CoV-2. Influenza caused by seasonal IAVs can be prevented by yearly vaccination and can be treated by FDA-approved anti-flu drugs, which are divided into four groups: M2 inhibitors, a PA inhibitor, a PB1 inhibitor and NA inhibitors [270]. Nonetheless, highly mutable IAVs that possess RNA genome segments and RNA polymerase without proofreading, different from the CoV RNA genome and RNA polymerase with proofreading, have continued to circulate in humans for more than one hundred years. Structural analysis, studies on receptor binding specificity and studies on inhibition of infection by synthetic sialylglycopolymers [19,271] have suggested that there is an invariant receptor binding site (RBS) on viral HA spikes that is critical for virus binding to a human-type receptor for infection. This easily reachable drug target that is abundant on the viral envelope is a promising target for the development of a universal and permanent anti-influenza drug against human-adapted viruses of H1–H16 HA subtypes. Recently, 6SLN-lipo PGA [270,271] was shown to be effective when administered alone and to have a synergistic effect when combined with an NAI drug against both pandemic and seasonal influenza viruses. In the case of CoVs, the RBS could be a potential drug target. However, CoVs recognize different receptors including protein receptors, CEACAM1a, NCAM, DDP4, APN and ACE2, and saccharide receptors, sialylated or non-sialylated saccharides. Some CoVs also recognize a co-receptor/attachment factor by a different binding pocket of the S protein and some βCoVs also carry an HE spike protein binding to an *O*-acetylated Sia receptor (Table 2). Thus, for the design of an anti-CoV against the RBS, consideration must be given to the role and importance of each receptor and to the use of two inhibitors in combination or the possibility of generating a single compound with two different inhibitory sites. 

Finally, studies to understand how human viruses emerge, to understand the pathogenesis of diseases, and to produce effective, safe and permanent (if possible) vaccines/drugs are essential for virus control and eradication. However, the most important thing is creating awareness about the results of human intrusion and disturbance of wildlife in order to minimize or eliminate direct contact of domestic animals/humans with wild animals for prevention of the next emerging disease.

## Figures and Tables

**Table 1:** Host range, viral receptor-binding glycoproteins and host receptors of influenza viruses.

Family	Genus	Genome	Type		Host	IAV Subtype or IBV^1^/ICV/IDV Lineage	Disease	Receptor-binding Protein	Host Receptor	Ref.
				Birds	Wild birds (frequently found in waterfowls and shorebirds)	H1–H16 and N1–N9 HP: H5NX, X = NA subtypes	No or mild disease HP: isolated from dead wild birds	HA	Terminal Neu5Acα2,3Gal Some gull/tern H16: α2,6Neu5Ac ≥ α2,3Neu5Ac Internal Duck type: non-fucosylate, SiaLe^c^, 3′SLN Gull type: fucosylated, SiaLe^x^ and 6-sulfo-SiaLe^x^ Gull/tern H16: 6′SLDN ≥ LSTb, but some 6-sulfo-SiaLe^x^ > 6′SLDN, LSTb	[23,24]
					Domestic birds (poultry)	H1–H13, N1–N9	LP: mild disease HP: multi-organ systemic disease	HA	Terminal Neu5Acα2,3Gal Internal HP higher affinity than LP: 3′SLN, SiaLe^c^ All LP and HP: 3′SLN > SiaLe^x^, SiaLe^c^ > SiaLe^a^, 3′SLN ~ SiaLe^c^ H5 poultry: 6-sulfo-3′SLN H7H7 from all species: 6-sulfo-3′SLN, 6-sulfo-SiaLe^x^ > nonsulfated SGPs	[25,26,27,28,29,30,31,32,33,34,35,36,37]
					Domestic ducks	HP: H5N1, H5N2, H5N3, H5N5, H5N6, H5N8, H7N2, H7N9				
					Other poultry	HP: H5N1, H5N2, H5N3, H5N6, H5N8, H5N9, H7N1, H7N2, H7N3, H7N4, H7N7, H7N9				
	*Alphainfluenzavirus*			Other animals	Pigs	H1N1, H1N2, H2N3, H3N1, H3N2, HPH5N1	Respiratory disease	HA	Neu5Acα2,6Gal > Neu5Acα2,3Gal Neu5AcGM3, Neu5GcGM3	[4] [38,39,40,41]
					Horses	H7N7, H3N8	Respiratory disease	HA	Mainly Neu5Gcα2,3Gal, Sialyllacto-*N*-tetraose a, SiaLe^x^	[42,43]
		8 (−)ssRNA segments			Dogs	H3N8, H3N2, H3N1, H5N2, HPAI H5N1, H1N1	Respiratory disease	HA	Canine H3N8: 6-sulfo-3′SLN and Neu5Acα2,3Galβ-	[38] [43,44,45,46]
					Cats	H7N2, H3N2, HPH5N1	Respiratory disease	HA	Feline H7N2: Siaα2,3Gal	[38,47] [48]
			A		Ferrets (a model for human infection and transmission)	Triple reassortant H1N1 SIV, A(H1N1)pdm09	Respiratory disease	HA	Expected to bind to Neu5Acα2,6Gal	[49,50]
					Occasionally in numerous species of mammals such as whales, seals, tigers, and minks	Seal H7N7 & whale H13N9 Tiger HPH5N1 Mink H9N2	Respiratory disease	HA	Seal H7N7 & whale H13N9: Siaα2,3Gal Tiger HPH5N1 HA with Q226 and G228 (H3 numbering): predicted to prefer Siaα2,3Gal Mink H9N2 HA with 226L: increased binding to Siaα2,6Gal	[51,52,53,54,55,56]
*Orthomyxoviridae*				Zoonosis	Poultry, pigs and other animals	H1, H2, H3, H5 (HP & LP), H6, H7 (HP & LP), H9, H10	HP: severe systemic disease Other zoonotic infections: ranging from mild upper respiratory tract infection to severe pneumonia and death. Possible to cause conjunctivitis, GI symptoms, encephalitis and encephalopathy	HA	Most still retains previous host-receptor specificity	[16] [57,58,59]
				Humans		1918-derived H1N1 (extinct), 1957-derived H2N2 (extinct), 1968-derived H3N2, 2009-derived H1N1	Pandemic and continued as seasonal flu: Primarily cause respiratory disease (usually pdm more severe, including pneumonia)	HA	Terminal Neu5Acα2,6Gal Internal 6′S(LN)_n_ Early human-adapted viruses: n = 1, 2, 3, etc. Long-term circulating viruses: n ≥ 3	[16,19] [60,61]
				Bats	Fruit-eating bats in Guatemala	Sl-BatH17N10 Fruit-eating bats in Peru: Ap-batH18N11	H17: liver, intestine, lung and kidney tissues, and rectal swabs H18: intestine tissue and rectal swabs	HA	MHC-II HLA-DR	[62,63]
					Fruit-eating bats in Egypt	Distinct from all known IAVs. HAs, closest to mallard H9 viruses with 73% aa identity	Viruses in bat oral swabs > in rectal swabs Able to replicate in mouse lungs	HA	Terminal Neu5Acα2,3Gal > Neu5Acα2,6Gal	[64]
	*Betainfluenzavirus*	8 (-)ssRNA segments	B	Humans, sporadically in seals, pigs, horses, pheasants and dogs.		Influenza B viruses (IBVs) 2 lineages - Victoria lineage B/Victoria/2/87-like viruses - Yamagata lineage: B/Yamagata/16/88-like viruses	Seasonal flu	HA	Wild types of both Yamagata (HA with F95 and N194) and Victoria (HA with G141, R162 and D196) lineages: Neu5Acα2,6Gal>Neu5Acα2,3Gal 2001–2007 clinical isolates in Taiwan: 83% of Yamagata-like strains prefer α2,6Sia; 17% prefer both α2,3Sia and α2,6Sia. 54% of Victoria-like strains prefer both α2,3Sia and α2,6Sia; 25% prefer sulfated glycan, either β-Gal-3-sulfate or 6-HSO_3_-Galβ1,4GlcNAc; 21% prefer α2,6Sia. Dual α2,3Sia and α2,6Sia-binding viruses seem to be associated with bronchopneumonia and gastrointestinal symptoms.	[65,66,67,68]
	*Gammainfluenzavirus*	7 (−)ssRNA segments	C	Humans (particularly in infants and children), pigs, cattle		Influenza C viruses (ICVs) 6 lineages: C/Taylor, C/Mississippi, C/Aichi, C/Yamagata, C/Kanagawa and C/Sao Paulo	Usually mild flu	HEF	Terminal C/Johannesburg/1/66 (C/Aichi lineage): Neu5,9Ac_2_α2,6Gal	[69,70,71,72]
	*Deltainfluenzavirus*	7 (−)ssRNA segments	D	Pigs, cattle, 91% seropositivity found among persons working with cattle		Influenza D viruses (IDVs) 3 lineages: D/OK, D/660 and D/Japanese D/Japanese lineage contains 2 sublineages: D/Yama2016 and D/Yama2019	Usually mild flu	HEF	Terminal D/OK: Neu5,9Ac_2_ and Neu5Gc9Ac either linked to α2,6Gal or α2,3Gal D/660: Neu5,9Ac_2_α2,6Gal, Neu5Gc9Acα2,6Gal and Neu5Gc9Acα2,3Gal	[15,72] [73]

3′SLN: 3′sialyllactosamine (Neu5Acα2,3Galβ1,4GlcNAcβ1); 6′SLDN: Neu5Acα2,6GalNAcβ1,4GlcNAcβ1; GI: gastrointestinal; HA: hemagglutinin; HEF: hemagglutinin-esterase-fusion; HLA-DR: human leukocyte antigen DR isotype; HP: highly pathogenic virus; IAV: influenza A virus; IBV^1^: influenza B virus; ICV: influenza C virus; IDV: influenza D virus; LP: low pathogenic virus; LSTb: lactoseries tetrasaccharide b (Galβ1,3(Neu5Acα2,6)GlcNAcβ1,3Galβ1,4Glcβ1); MHC-II: major histocompatibility complex II; NA: neuraminidase; Neu5,9Ac_2_: 5-*N*-acetyl-9-*O*-acetylneuraminic acid; Neu5Ac: 5-*N*-acetylneuraminic acid; Neu5AcGM3: Neu5Acα2,3Galβ1,4Glcβ1-1′Ceramide; Neu5Gc: 5-*N*-glycolylneuraminic acid; Neu5GcGM3: Neu5Gcα2,3Galβ1,4Glcβ1-1′Ceramide; Sia: sialic acid; SiaLe^a^: sialyl-Lewis^a^ (Neu5Acα2,3Galβ1,3(Fucα1,4)GlcNAcβ1); SiaLe^c^: sialyl-Lewis^c^ (Neu5Acα2,3Galβ1,3GlcNAcβ1); SiaLe^x^: sialyl-Lewis^x^ (Neu5Acα2,3Galβ1,4(Fucα1,3)GlcNAcβ1); Sialyllacto-*N*-tetraose a: Neu5Acα2,3Galβ1,3GlcNAcβ1,3Galβ1-4Glcβ1; SIV: swine influenza virus.

**Table 2:** Host range, viral receptor-binding glycoproteins and host receptors of coronaviruses.

Family	Subfamily	Genus	Genome	Lineage	Host	CoV	Disease	Receptor-binding Protein	Host Receptor		Ref.
									Primary Receptor	Attachment Factor	
		*Alphacoronavirus* (gr. 1)	Single linear (+)ssRNA		Bats	Ro(HKU10)-, Hi(HKU10)-, Rh(HKU2)-, Sc(512)-, Mi(1A, 1B, HKU8)- BatCoV	Unknown				[74]
					Other animals	FeCoV I & II (nonvirulent form of FCoV), CCoV, TGEV, PEDV	Gastroenteritis	S1	S1^B^ (S1-CTD) Aminopeptidase N (APN; CD13), not for PEDV and FeCoV I	S1^A^ (S1-NTD) α2,3Neu5Gc >Neu5Ac (TGEV traveling to a target site), Neu5Ac > Neu5Gc (PEDV)	[75,76,77,78,79,80]
						FIPV (virulent form of FCoV)	Peritonitis, severe wasting	S1	fAPN for type II, not type I FIPV	Coreceptor for both types I and II: DC-SIGN	[81,82]
						PRCoV	Respiratory disease	S1-CTD	pAPN (res. 366–369, 727–790)		[83]
					Humans	HCoV-229E, HCoV-NL63	Generally URTI	S1-CTD	NL63: hACE2 229E: hAPN (res. 208, 241–319)		[84]
					Rodents	ChRCoV (HKU24)	Unknown				[85]
						RCoV	Respiratory disease, sialodacryoadenitis	HE	Unknown	HE (type II) 4-*O*-Ac-Sias, 4,5-di-*N*-acetylneuraminic acid α-methylglycoside (α-4-*N*-Ac-Sia)	[86,87]
						MHV	Gastroenteritis, hepatitis, encephalomyelitis	S1-NTD, HE	S1-NTD Murine CEACAM1	HE Strain DVIM: 9-*O*-Ac-Sias Strains S, JHM: 4-*O*-Ac-Sias	[88,89]
				A	Other animals	BCoV, EqCoV(ECoV)	Enteritis, respiratory disease	S1-NTD, HE	S1-NTD 9-*O*-acetylated sialoglycans (unknown for EqCoV)	HE (type I) 9-*O*-acetylated sialoglycans, 7,9-di-*O*-acetyl Sia	[86,90]
						PHEV	Vomiting, wasting, encephalomyelitis	S1, HE	S1 (res. 291–548) Porcine NCAM S1-NTD 9-*O*-Ac-Sia	HE 9-*O*-Ac-Sias	[91,92,93]
					Humans	HCoV-OC43, HCoV-HKU1	Generally URTI	S1-NTD, HE	S1-NTD Neu5,9Ac_2_	HE Neu5,9Ac_2_ Progressive loss of binding during circulation in humans OC43: accumulation of mutations HKU1: massive deletions	[92,94]
					Bats	SARSr-Rh(HKU3)-, SARSr-Rp-, SARSr-Rs-, SARSr-Rf-BatCoV, Bat-SL-RaTG13, Bat-SL-RmYN02	Viruses in anal/fecal swabs		RsACE2, 3 aa substitutions in RpACE2: shown to be used by SARS-CoV		[95,96,97]
		*Betacoronavirus* (gr. 2)	Single linear (+)ssRNA		Other animals	Masked palm civet, raccoon dog: SARS-CoV-like virus SARS-CoV real-time RT-PCR-positive animals: red fox, Sikkim rat, wild boar, etc. Pangolin: SARS-CoV-2-like CoV SARS-CoV-2 RT-PCR-positive animals: dog, cat, tiger, etc.	Palm civets: viruses in rectal and/or throat swabs Pangolins: viruses from lung tissues Domestic cats, dogs: viruses in oral/nasal specimens	S1-CTD	Host ACE2 Prototype: viral S gene has no SNV.		[98,99,100,101,102,103]
*Coronaviridae*	*Orthocoronavirinae*			B	Zoonosis	SARS-CoV	SARS in humans: atypical pneumonia (type I pneumocytes), ARDS, gastroenteritis	S1	S1-CTD hACE2 - Nov 2002–Jan 2003 (early epidemic, HP, animal/human-to-human by close contact): viral S gene contains 17–22 SNVs. - Feb–Jul 2003 (epidemic, HP, human-to-human by close contact): 25–27 SNVs - Dec 2003–May 2004 (re-appearance, LP, animal-to-human): 2–7 SNVs	DC-SIGN, DC-SIGNR and LSECtin: their roles are less clear.	[100] [104] [105]
					Pandemic	SARS-CoV-2	COVID-19: URTI, LRTI (non-life-threatening pneumonia, severe pneumonia with ARDS)	S1-CTD	hACE2 (efficient and sustained spread among humans)		[9]
					Bats	Ty(HKU4)-, Hp(HKU25)-, Pi(HKU5)-BatCoV, NeoCoV	Virus in feces	S1	S1-CTD Ty- and Hp-BatCoVs, but not Pi-BatCoV, can bind to hDPP4.	S1-NTD Binds to the intestinal epithelium in common pipistrelle bats but not in serotine bats and frugivorous bats.	[106,107,108]
				C	Dromedary camels	dromedary MERS-CoV	Virus/RNA in nasal swabs and lung tissue samples. An experimental study: mild upper respiratory tract disease	S1	S1-CTD dromedary DDP4 found in various organs, particularly in nasal turbinate, trachea and lungs	S1-NTD α2,3-sialic acid The S1^A^ domain binds to the dromedary nasal epithelium but not to the porcine/rabbit nasal epithelium.	[108,109,110,111]
					Zoonosis	MERS-CoV	Atypical pneumonia	S1	S1-CTD hDDP4 (CD26) Note: Intercellular spread of the virus through cell-cell fusion does not require DDP4 receptors but requires TMPRSS2 activity.	S1-NTD Short and long α2,3Neu5Ac > α2,6Neu5Ac glycans The S1^A^ domain binds to the human alveolar epithelium (type II pneumocytes).	[104] [108] [112] [113]
				D	Bats	Ro-BatCoV (HKU9)	Unknown				[74]
		*Gammacoronavirus* (gr.3)	Single linear (+)ssRNA		Birds	Infectious bronchitis virus (IBV^2^)	Bronchitis, nephritis, reproductive problems	S1-NTD	IBV: α2,3-linked sialic acids type 1 lactosamines IBV strain Beaudette: heparan sulfate		[114] [115]
						TuCoV(TCoV), GfCoV	Enteritis	S1	Nonsialylated type 2 poly-*N*-acetyl-lactosamines		[114]
		*Deltacoronavirus* (a new group)	Single linear (+)ssRNA		Birds	FalCoV, HouCoV, PiCoV	Unknown				[116]
					Other animals	PorCoV (PDCoV)	Diarrhea in newborn piglets	S1	pAPN in porcine alveolar macrophages but not necessary for infection of lung-derived fibroblast cells		[77]

9-*O*-acetyl-sialic acid; aa: amino acid; ACE2: angiotensin-converting enzyme 2; Ap-: *Artibeus planirostris*; APN: aminopeptidase N; ARDS: acute respiratory distress syndrome; Bat-SL-RaTG13: SARS-like CoV isolated from *Rhinolophus affinis* (Ra); Bat-SL-RmYN02: SARS-like CoV number 02 isolated from *Rhinolophus malayanus* (Rm) in China’s Yunnan (YN) province; BCoV: bovine coronavirus; CCoV: canine coronavirus; CEACAM1: carcinoembryonic antigen-related cell adhesion molecule 1; ChRCoV: China Rattus coronavirus; CoV: coronavirus; CTD: C-terminal domain; DC-SIGN: dendritic cell-specific ICAM-3 grabbing non-integrin; DC-SIGNR: DC-SIGN-related protein; EqCoV (ECoV): equine coronavirus; FalCoV: falcon coronavirus; FCoV: feline coronavirus; FeCoV I & II: feline coronavirus type I & II; FIPV: feline infectious peritonitis; GfCoV: guineafowl coronavirus; HCoV: human coronavirus; HE: hemagglutinin-esterase; Hi-: *Hipposideros* bat; HouCoV: houbara bustard coronavirus; Hp-: Chinese pipistrelle bat (*Hypsugo pulveratus*); IBV^2^: infectious bronchitis virus; LRTI: lower respiratory tract infection; LSECtin: liver and lymph node sinusoidal endothelial cell C-type lectin; MERS-CoV: Middle East respiratory syndrome-coronavirus; MHV: mouse hepatitis virus; Mi-: *Miniopterus* bat; NCAM: neural cell adhesion molecule; Neo-: *Neoromicia capensis*; NTD: N-terminal domain; PEDV: porcine epidemic diarrhea virus; PHEV: porcine hemagglutinating encephalomyelitis virus; Pi-: *Pipistrellus* bat; PiCoV: pigeon coronavirus; PorCoV (PDCoV): Porcine deltacoronavirus; PRCoV: porcine respiratory coronavirus; RCoV: rat coronavirus; Rf-: *Rhinolophus* *ferrumequinum*; Rh-: *Rhinolophus* bat; Ro-: *Rousettus* bat; Rp-: *Rhinolophus pusillus*; Rs-: *Rhinolophus* *sinicus*; S1: receptor-binding subunit of spike (S) protein; SARS-CoV: severe acute respiratory syndrome coronavirus; SARS-CoV-2: severe acute respiratory syndrome coronavirus-2; SARSr-CoV: SARS-related coronavirus; Sc-: *Scotophilus* bat; Sl-: *Sturnira lilium*; SNVs: single nucleotide variants; TGEV: transmissible gastroenteritis virus; TuCoV(TCoV): turkey coronavirus; Ty-: *Tylonycteris* bat; URTI: upper respiratory tract infection.
